# Emergent spatiotemporal population dynamics with cell-length control of synthetic microbial consortia

**DOI:** 10.1371/journal.pcbi.1009381

**Published:** 2021-09-22

**Authors:** James J. Winkle, Bhargav R. Karamched, Matthew R. Bennett, William Ott, Krešimir Josić

**Affiliations:** 1 Department of Mathematics, University of Houston, Houston, Texas, United States of America; 2 Department of Mathematics, Florida State University, Tallahassee, Florida, United States of America; 3 Institute of Molecular Biophysics, Florida State University, Tallahassee, Florida, United States of America; 4 Department of Bioengineering, Rice University, Houston, Texas, United States of America; 5 Department of Biosciences, Rice University, Houston, Texas, United States of America; 6 Department of Biology and Biochemistry, University of Houston, Houston, Texas, United States of America; Duke University, UNITED STATES

## Abstract

The increased complexity of synthetic microbial biocircuits highlights the need for distributed cell functionality due to concomitant increases in metabolic and regulatory burdens imposed on single-strain topologies. Distributed systems, however, introduce additional challenges since consortium composition and spatiotemporal dynamics of constituent strains must be robustly controlled to achieve desired circuit behaviors. Here, we address these challenges with a modeling-based investigation of emergent spatiotemporal population dynamics using cell-length control in monolayer, two-strain bacterial consortia. We demonstrate that with dynamic control of a strain’s division length, nematic cell alignment in close-packed monolayers can be destabilized. We find that this destabilization confers an emergent, competitive advantage to smaller-length strains—but by mechanisms that differ depending on the spatial patterns of the population. We used complementary modeling approaches to elucidate underlying mechanisms: an agent-based model to simulate detailed mechanical and signaling interactions between the competing strains, and a reductive, stochastic lattice model to represent cell-cell interactions with a single rotational parameter. Our modeling suggests that spatial strain-fraction oscillations can be generated when cell-length control is coupled to quorum-sensing signaling in negative feedback topologies. Our research employs novel methods of population control and points the way to programming strain fraction dynamics in consortial synthetic biology.

## Author summary

Synthetic microbial collectives are more versatile and robust than single strain populations. However, the function of such collectives is sensitive to their spatiotemporal organization. Here, we show that the spatiotemporal composition of synthetic microbial consortia can be controlled by dynamically modulating the average cell length of constituent strains. We show that such modulation can confer an emergent “mechanical fitness” advantage upon the shorter length strain. We use both a biophysically realistic agent-based model to test the impact of cell shape on spatiotemporal dynamics, and a conceptually simpler stochastic lattice model to explain the essential mechanisms driving the dynamics.

## Introduction

Understanding and designing microbial consortia with distributed functionality is of increasing interest in synthetic biology [1–7]. Assigning different functions to genetically distinct strains in a bacterial consortium reduces the metabolic load on each strain and thus allows for more complex functionality and greater robustness [8–12]. Synthetic gene circuits previously engineered in single strains, such as feedback oscillators and toggle switches [13, 14], have recently been implemented in consortia [10, 12, 15, 16]. However, we still lack the mathematical and computational tools that would allow us to engineer such systems in a principled way.

Synthetic microbial consortia intrinsically require balance and control of population strain fractions to achieve desired genetic circuit functionality. Important population control studies of synthetic bacterial collectives in the literature have employed both theoretical and experimental approaches, and a number of different control mechanisms have been introduced. Such approaches include predator-prey systems [17], cross-feeding auxotrophs [18], toxin-antitoxin [19] and “ortholysis” [1] mechanisms, and external switching control [20]. Although many of these methods employ regulating feedback, population dynamics achieved via toxic agents released by a cell must be tightly controlled to prevent unwanted expression and non-robust population behaviors. Studies that focus on distributed microbial systems have also been wide-ranging and include those of information exchange between constituent strains [21], ecological dynamics [22–24], metabolic resource allocation [25, 26], microbial social interactions [27], and the human microbiome [28, 29]. In each of these examples, balance and control of constituent parts is central to robust functionality [30, 31].

In contrast to population control mechanisms employing a toxin, here we suggest that the strain fraction in a microbial consortium can be controlled by changing the average division length of cells within each strain. Our approach draws inspiration from two active research areas: The first area focuses on how cell aspect ratio (cell length divided by cell width) affects cell ordering in close-packed environments [32–37]. Previous studies have combined experimental, theoretical, and computational approaches to demonstrate that decreasing cell length generally decreases cell nematic ordering in spatially confined environments such as monolayer microfluidic devices (the term *nematic order* is a measure of the local alignment of apolar, rod-like shapes such as bacteria [32, 35, 36, 38, 39] and is borrowed from the study of liquid-crystals [40, 41]). The second research area concerns *programming* bacterial cell aspect ratio by modulating expression of the cell-division proteins MreB and FtsZ [42–44]. Our modeling approach explores a synthesis of these two lines of research by proposing that cell division length can be modulated dynamically in a single experiment.

We simulated the growth, mechanical interactions, and intercellular signaling of two microbial strains with different average cell lengths in a spatially-extended, monolayer microfluidic device. We considered populations of rod-shaped bacteria whose axial growth leads to emergent columnar population structures [33, 45], and found that decreasing a cell’s average length can alter population dynamics by destabilizing this emergent columnar organization.

Using an agent-based model (ABM), we found that this mechanism gave shorter cells a competitive advantage (“mechanical fitness”) in close-packed microfluidic trap simulations. By *mechanical fitness* we mean the advantage a strain has over others due to the *physical* properties of the cells, and *physical* interactions with its environment. In particular, we propose here that a shorter average cell length can provide a competitive advantage to a strain co-cultured with longer cells by allowing shorter cells to displace other cells from a domain, thus increasing their ability to *compete* [46] due to mechanical properties. To better understand the essential dynamical mechanisms behind the emergent dynamics, we also developed a complementary lattice model (LM) based on the key features of microbial growth and interactions [33]. We mapped the cell division length to a single parameter, the probability of cell rotation upon division, which served as proxy for cell size. Simulations of the LM confirmed that rotation probability controlled the level of disorder in the population and allowed us to confirm the mechanism by which the more disordered strain displaces its counterpart. Finally, we used the ABM with cell-length control coupled to quorum-sensing signaling to generate spatiotemporal population oscillations.

## Results

We hypothesized that average cell length of capsule-shaped bacteria, such as *E.coli*, can affect local cell ordering and relative fitness in a confined environment. To test this hypothesis, we modeled a population of bacteria whose division length can be modulated via an external signal in real time (within one generation).

### Agent-based modeling

#### Single-strain nematic cell ordering

**Fig 1:**
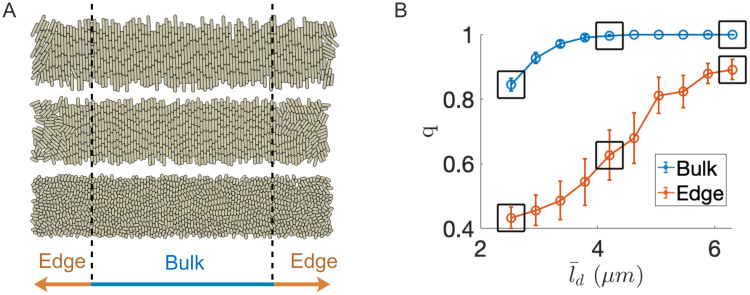
Average cell length affects nematic ordering in open-walled microfluidic trap simulations. (A) ABM simulation snapshots for three different average cell division lengths (2.5, 4.2, and 6.3 *μ*m, bottom to top) in a 20 x 100 *μ*m open-walled microfluidic trap. For each simulation snapshot the order parameter *q* (Eq (1)) was measured in two different trap regions once the population reached steady-state. (B) Order parameter *q* in the bulk and edge subregions. Circles and error bars represent means and standard deviations, respectively, for a single, representative simulation at each division length. Data was sampled twice per generation for 120 total samples (≈ 20 hours) after population stabilization (≈ 5 hours after cell seeding). With decreased average division length, cell ordering decreased in both regions, but disorder persisted in the edge regions even for the longest division lengths simulated. Boxed data corresponded to the three cases shown in panel (a).

We first used an agent-based model (ABM) of a single strain of *E.coli* growing and dividing in an open-walled microfluidic device (see Methods) to relate average cell length to steady-state cell ordering in two regions of the trap (bulk and edge; See Fig 1). Each simulation was initiated by seeding the trap with 32 cells of each strain, randomly placed within the trapping region [45]. Following a trap-filling transient, cells in the bulk region formed nematicaly aligned vertical columns, as previously reported in modeling and experimental studies [15, 32, 33, 35, 47, 48]. This bulk alignment of cells is driven by asymmetry of the trap geometry: Cells grow toward the nearest boundary to minimize inhibition to their growth and division [32, 33]. This results in the approximately vertical alignment of cells in the bulk of rectangular domains, which is driven by the anisotropic stress tensor of the local population [35]. Near the left and right boundaries, the closest boundary is in transition and the cells exhibit less alignment.

We found that the ordering of cells in each region also depended on the average division length, ${\bar{l}}_{d}$, of the cell: Ordering in both regions decreased with decreasing ${\bar{l}}_{d}$ (Fig 1B), but maximal ordering in the bulk region required a minimum ${\bar{l}}_{d}$. We define the order parameter as

$$\begin{array}{c} q=\sqrt{{\left\langle \cos2\phi \right\rangle }^{2}+{\left\langle \sin2\phi \right\rangle }^{2}}, \end{array}$$ (1)

where *ϕ* denotes cell angle from horizontal in the lab frame and 〈⋅〉 signifies a region (bulk or edge) average. Correlation between cell length and nematic ordering has been reported previously [34, 37, 49].

For the smallest average division length we simulated (2.5 *μ*m, Fig 1A, bottom panel), the bulk population did not exhibit complete disorder (i.e., *q* > 0, where *q* = 0 represents a completely disordered, random distribution of cell orientations [49]). However, we still observed cells that were oriented *horizontally* at random times and locations in each simulation run. We also observed horizontal cells in the bulk region for larger values of ${\bar{l}}_{d}$, but not when ${\bar{l}}_{d}$ was greater than approximately 4 *μ*m. Horizontally oriented cells are significant because they can invade an adjoining column by axial growth (see S3 Fig). Based on these results, we hypothesized that horizontally oriented cells in the bulk region of a microfluidic population can alter population dynamics in two-strain consortia if one strain’s average division length is sufficiently small (${\bar{l}}_{d}<4\;\mu \text{m}$) compared to the other (${\bar{l}}_{d}>4\;\mu \text{m}$), assuming a constant cell width of 1 *μ*m.

In computational and experimental studies of morphologically homogeneous multi-strain populations, columnar cell structure (nematic order) leads to long-term stabilization of the strain fractions [45, 48]. We thus conjectured that emergent nematic *disorder* could result in *destabilization* of columnar structure and thereby affect strain fraction stability.

**Fig 2:**
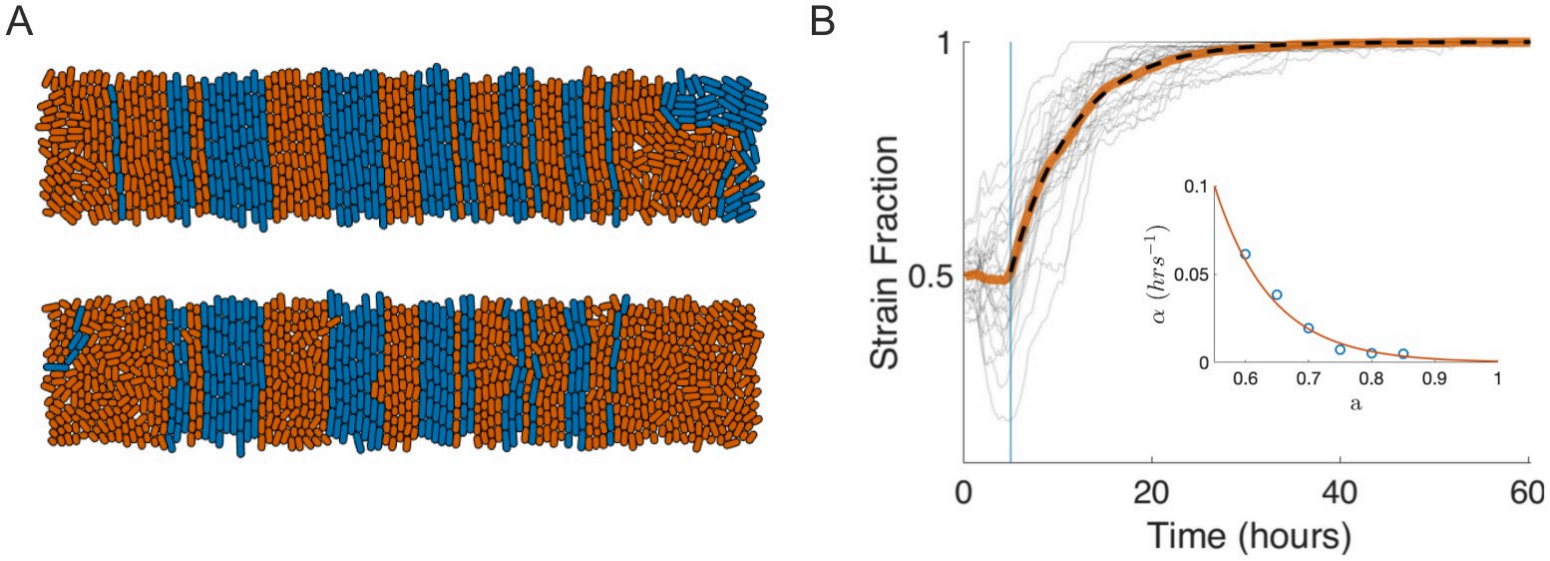
Cell morphology drives consortial population dynamics and strain fixation. (A) Two-strain ABM simulation snaphots in a 20 x 100 *μ*m trap. Top panel: With equal average cell division lengths (${\bar{l}}_{d}=4.2\;\mu \text{m}$), the consortium exhibited emergent columnar structure and stable strain population fractions in the bulk, as in Fig 1. Bottom panel: A snapshot taken approximately 5 generations (1.5 hours) after the mutant (orange) strain’s average division length was reduced by a factor of *a* = 0.6 (resulting ${\bar{l}}_{d}=2.5\;\mu \text{m}$). The increased rotational propensity of the smaller-length orange strain led to ejection of the WT (blue) strain by lateral invasion of its columns and subsequent growth-induced cell flow toward the open boundaries. Visible in lower panel are horizontal mutant cells beginning to destabilize adjacent WT columns. (B) Mutant strain fraction increased over time due to columnar invasion of the WT strain. At *t* ≥ 5 hours (blue line), the mutant strain’s average division length was reduced by factor *a* = 0.6. Destabilization of the columnar structure of the WT strain led to its eventual extinction in all simulations; grey curves: 20 individual simulations; solid orange curve: mean strain fraction trajectory; dashed curve: Fit of 1 − 0.5*e*^−*α*(*t*−5)^. Inset: The fit rate parameter *α* decreased with the length-reduction factor *a*.

**Fig 3:**
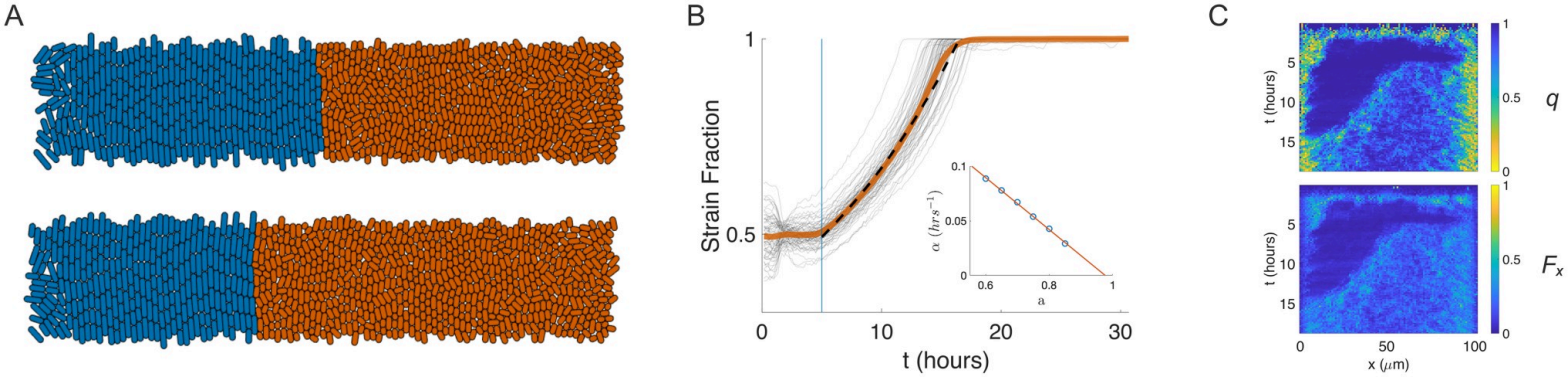
Single-interface consortial population dynamics and bulk forcing. (A) Snapshots of two-strain ABM simulation with cell strains seeded in separate halves of the trap. As in Fig 2, we reduced the mean division length of the mutant cells to ${\bar{l}}_{d}=2.5\;\mu \text{m}$ (*a* = 0.6) after stabilization of the population and emergence of nematic order in the bulk. Snapshots correspond to (top) one generation and (bottom) 10 generations (≈ 3 hours) after induction. (B) Mutant strain fraction time series for 20 ABM simulations. In contrast to the invasion mechanism illustrated in Fig 2, the mutant strain here acted cooperatively by “bulk forcing”: The WT strain bulk population was pushed laterally and ejected out the left, open trap boundary. Grey curves: 20 individual simulations; solid orange curve: mean strain fraction trajectory; dashed curve: fit of 0.5*e*^*α*(*t*−5)^. Inset: the fit parameter *α* vs. the division length scale parameter, *a*. (C) Mechanisms of the bulk forcing are revealed by kymographs. Top panel: The order parameter, *q*, averaged over 1*μ*m columns; Bottom panel: Growth-expansion force, *F*_*k*_, for each cell was projected horizontally to compute $F_{x}\left(x\right)≔\frac{1}{n}\sum_{k=1}^{n}F_{k}\cos\phi_{k}$, where *ϕ*_*k*_ is the cell angle from horizontal and the average is taken over cells *k* in a 1 *μ*m column *x*.

To test this hypothesis, we performed ABM simulations of two-strain bacterial consortia where one strain’s average division length was reduced after the population structure stabilized with nematic order. We chose a ‘wild-type’ (WT) average division length of 4.2 *μ*m. For this division length we observed nearly complete nematic ordering (*q* ≈ 1) in the bulk of the population in steady state in single-strain simulations (see Fig 1). We performed simulations with two strains and with two initial conditions: We used the WT strain and a mutant strain (whose average cell length was reduced after population stabilization) in both banded (Fig 2) and strain-separated (Fig 3) initial conditions.

#### Well-mixed population bands and column invasion

In our first two-strain simulations, we seeded the trap with a total of 64, randomly placed, WT (blue) and mutant (orange) cells, where each strain type was selected with equal probability (see Fig 2B and S1 Movie). The two strains differed only in their average division lengths (see Methods). The two strains had identical growth rates: The rate at which the length of each cell grew was proportional to the cell’s length. As the width of each cell was constant, this means that rate at which a population’s volume increased was independent of the average length of cells in the population, or their divison length [50].

In the initial transient period of seed-cell growth and expansion (*t* < 5 hours), the two strains’ division lengths were identical. During this initial period, strain fractions stabilized after the formation of single-strain, columnar bands of various widths [45, 48] (see Fig 2A, top image, and S1 Movie). With this emergent, nematic cell ordering, strain identity of each column was determined by the anchoring mother-cell position, which was located at or near the center of the column [45, 48].

At *t* = 5 hours (approximately 15 cell generations), we reduced the mutant strain’s average division length by a factor of *a* = 0.6, and this reduction persisted through the remainder of the simulation. As a result of the division length decrease, nematic ordering markedly decreased for the mutant strain (Fig 2A, bottom image), and as predicted by our single-strain simulations, mutant cells randomly rotated into horizontal orientations. We then observed that horizontally oriented cells *invaded* the adjoining columns of the WT strain. When a cell rotated into an adjoining mother-cell position, subsequent growth and division of the invading cell allowed the invader and its descendants to occupy the invaded column by occupying the mother-cell position in the center of the column.

Fig 2B shows the resulting temporal evolution of the mutant strain fraction for 20 ABM simulations with a random cell-seeding condition, and for *a* = 0.6. We found that an exponential function of the form *f*(*t*) = 1 − 0.5*e*^−*α*(*t*−5)^ (dashed curve) provided an excellent description of the cell fraction (solid orange curve) average over these simulations. The observed exponential decrease in WT strain fraction has an intuitive explanation: If we view the loss of WT columns as a stochastic death process and assume that mutant cells invade WT bands at a constant rate *per WT band*, then the death rate is proportional to the number of remaining WT bands. We additionally found that the computed rate parameter for the exponential fit, *α*, itself depended exponentially on the average division length reduction factor, *a*. In the inset to Fig 2B, the exponential (orange curve) was fit to values of the rate parameter *α* (blue circles) computed from similar ABM simulations per value of *a* (see complete data in S1 Fig and S2 Movie). As expected from the simulations shown in Fig 1, *α* decreased as *a* increased due to decreased frequency of mutant cell rotations. However, why *α* depends exponentially on *a* remains to be explained.

#### Strain-separated populations and bulk rotational forcing

In the previous two-strain ABM simulations we used a random initial seeding in space, which resulted in a banded population structure at steady state [45]. To understand the effect of the initial spatial patterning of strains on their subsequent population dynamics, we seeded the trapping region so that the WT (blue) strain occupied the left half of the trap and the mutant (orange) strain the right half. This initializaton resulted in a single, initially stable strain interface near the center of the trap. However, in contrast to the previous ABM simulations, we did not observe frequent cell rotation and columnar invasion at the interface after we reduced the mutant strain’s average division length. Additionally, we observed that the mutant strain’s population growth rate *increased* over time (Fig 3B, compare Fig 2B). We conjectured that a different mechanism was responsible for the population dynamics in the strain-separated simulations than for those of the banded simulations.

As in the previous simulations, after cell-length reduction was induced in the mutant strain, nematic disorder in that strain increased (as measured by the order parameter *q*, see Fig 3C and S3 Movie). We observed that rather than rotating horizontally and invading the WT strain at the single interface, the mutant cells exerted a cooperative, horizontal force on the WT population due to the cumulative disorder exhibited in the *bulk* of mutant strain. We therefore hypothesized that the observed increased rate of ejection of the WT strain was due to the increased probability that a mutant cell would grow and divide in a non-vertical direction (i.e, not in a vertically aligned state) once the bulk disorder in that strain was established and that the observed cooperative horizontal force would be proportional to the size of the bulk mutant population. Hence, the force imbalance leads to displacement and ejection of the more ordered WT strain, and the resulting rate of ejection would increase in time due to positive feedback.

To confirm our hypothesis that this second mechanism was responsible for the population dynamics observed in the strain-separated initial condition, we measured both *q*, the order parameter, and *F*_*x*_, the column-averaged horizontal component of the cells’ growth-expansion force and plotted them in kymographs. We define $F_{x}\left(x\right)≔\frac{1}{n}\sum_{k=1}^{n}F_{k}\left(x\right)\cos\phi_{k}$, where *ϕ*_*k*_ is the long-axis cell angle from horizontal in the lab frame, *F*_*k*_ is the cell growth-expansion force [48], and we average over all cells *k* in a 1 *μ*m-wide column at horizontal location *x*. The decrease in *q*, following reduction of division length in the mutant strain, is strongly correlated with an increase in *F*_*x*_ in the bulk of the trap (Fig 3C and 3D). The resulting horizontal force imbalance between the two strains thus imparted a net horizontal force on the bulk population of the WT strain. The magnitude of this force was indeed proportional to the size of the mutant population, which resulted in positive feedback as originally hypothesized. We note that the observed persistent nematic disorder at the trap’s open boundaries are due to these boundaries being stress-free (see [48] and Fig 1).

We thus observed that population dynamics in strain-separated simulations differed from those of the banded simulations in ways that confirmed the existence of a different strain-displacement mechanism. Fig 3B shows the evolution of the mutant strain fraction again averaged over 20 ABM simulations (orange curve). An exponential, *f*(*t*) = 0.5*e*^*α*(*t*−5)^, (dashed curve) provided a good fit to this average, up until the ejection of the WT strain. This exponentially increasing trend in the fraction of the WT strain is consistent with the positive feedback mechanism we identified above. As we did for the banded simulations, we computed the rate parameter, *α*, for different values of the division-length scaling factor, *a* (Fig 3B, inset, circles and S4 Movie). In contrast to the banded simulations, the fit rate parameter, *α*, depended approximately linearly on *a* in the strain-separated case (complete simulation results are in S2 Fig).

Thus, two different initial seeding conditions in the trap resulted in the displacement of the WT strain from the trap but via two distinct mechanisms. However, both mechanisms could be present under both conditions. While it is easy to understand that bulk-forcing may cancel on average (see S2 Movie) with banded initial conditions, the absence of columnar invasions in the strain-separated simulations has several possible explanations. One reason is that with only a single interface, there are simply less chances for an invasion to occur. Another potential reason is a decreased compliance of the WT strain with invasion by the mutant strain with a single interface: Emergent motion of the interface boundary may help the WT boundary “escape” invasion events by transport at an advective timescale comparable to that of invasion events.

### Lattice model

The ABM captures growth, cell-cell mechanical interactions, and diffusive signaling in realistic detail. As a result, however, it has many degrees of freedom, which make observed behaviors difficult to analyze in isolation and computationally expensive to simulate across even a narrow parameter space for each independent variable. Consequently, we used a complementary lattice model (LM) to understand the essential mechanisms by which cell morphology drives the spatiotemporal dynamics of consortia (See Methods). Since individual cells in the LM were simulated on a regular lattice, we used the rotation probability, *p*_rot_, as a proxy of cell size: As in the ABM, we assumed that shorter cells are less likely to change orientation than their longer counterparts. ABM simulations showed that decreasing average cell division length confers a fitness advantage via two distinct mechanisms: columnar invasion and lateral bulk forcing. Our simplified LM incorporates only the essential features of the ABM allowing us to test the hypothesis that changes in rotation probability alone drive both invasion and forcing.

#### Impact of differing rotation probabilities in the LM

We hypothesized that assigning different rotation probabilities to the two strains in the LM would recapitulate the strain-strain interactions and the resulting spatiotemporal dynamics observed in the ABM. To verify this hypothesis, we considered two sets of LM simulations that mirrored those we performed with the ABM.

**Fig 4:**
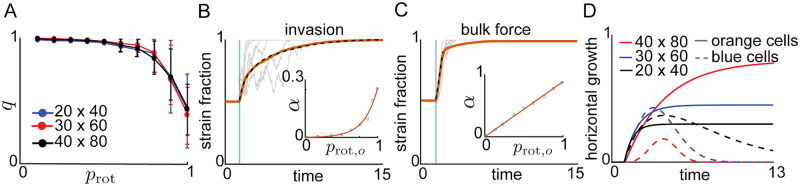
Lattice model simulations. (A) As in the ABM the order parameter, *q*, is obtained by averaging cell orientations across the trap at different times. Simulations with a single strain and different lattice sizes show that nematic disorder increases with *p*_rot_. (B) In banded LM simulation, we observed the same invasion mechanisms as in the ABM simulations. We initially set *p*_rot,*b*_(*t*) = *p*_rot,*o*_(*t*) = 0 (where *p*_rot,*k*_ denotes the probability of rotation for a cell of strain *k*) so that during a transient period the two strains formed alternating stripes. Setting *p*_rot,*b*_(*t*) = 0 and *p*_rot,*o*_(*t*) = 0.5 at *t* ⩾ 1 (light blue line), led to the ejection of the WT (blue) strain. The orange curve shows the evolution of the strain fraction averaged over 1000 trajectories, while grey curves show sample trajectories. The dashed curve shows the fit of the function 1 − 0.5*e*^−*α*(*t*−1)^ to the average mutant (orange) strain fraction. Inset: The rate parameter, *α*, as a function of *p*_rot,*o*_ (circles represent LM data, compare to inset in Fig 2B). (C) The LM also captured the bulk forcing mechanism we observed in the ABM. Simulations in (C) matched those in (B), with one change: We initially filled the left half of the lattice with mutant cells, and the right half with WT cells. We fit the temporal evolution of the average mutant strain fraction to 0.5*e*^*α*(*t*−1)^ over *t* ∈ [1, 2]. Inset: The rate parameter *α* depends linearly on *p*_rot,*o*_ (compare to inset in Fig 3B) (D) With a single interface between the strains, horizontally growing cells forced out cells of the opposite strain, equivalent to the bulk forcing mechanism in the ABM. We plot the temporal evolution of the mean horizontal growth propensities for three lattice sizes with *p*_rot,*b*_ = 0.1 and *p*_rot,*o*_ = 0.5. Simulations in (C) corresponded to the red curves.

Before performing two-strain LM simulations, we verified that tuning the rotational probability *p*_rot_ in isogenic LM simulations reproduced the increase in nematic disorder with a reduction in average cell division length observed in the ABM. Fig 4A shows that in the LM the order parameter, *q*, computed as in the ABM, decreased, and cells were more likely to be oriented horizontally with an increase in *p*_rot_ (see S7 Fig).

Our first set of two-strain LM simulations mirrored the ABM simulations shown in Fig 2. We initialized each LM simulation by randomly assigning to each lattice site a cell of either the blue (WT) strain or the orange (mutant) strain. Initial cell orientations were random (see Methods and S6 Movie). During the first phase of the simulation, we set *p*_rot,*b*_ (blue strain) and *p*_rot,*o*_ (orange strain) to zero to allow single-strain bands of vertically oriented cells to emerge. At time *t* = 1, we set *p*_rot,*b*_ = 0 and *p*_rot,*o*_ = 0.5. Fig 4B shows the temporal evolution of the mutant strain fraction, averaged over 1000 LM simulations (orange curve). Similarly to the ABM fitting, the function *f*(*t*) = 1 − 0.5*e*^−*α*(*t*−1)^ closely fits this average temporal evolution. When fit to data, the rate *α* varied approximately exponentially with *p*_rot,*o*_ (Fig 4B, inset, and S8 Fig). The LM mutant strain fraction dynamics and the dependence of *α* on *p*_rot,*o*_ closely matched the ABM mutant strain fraction dynamics and the dependence of *α* on *a* in the ABM simulations. This is particularly evident when we plot *α* versus 1 − *p*_rot,*o*_ (compare Fig 2B and S9 Fig). This suggests that the LM successfully captures the invasion mechanism.

Our second set of two-strain LM simulations mirrored those of Fig 3. We initialized the lattice so that mutant cells occupied the left half of the trap and the WT cells occupied the right half of the trap, producing a single interface between the strains. To capture the decrease in division length upon induction, we set *p*_rot,*o*_ < *p*_rot,*b*_ at time *t* = 1. The increased rotational freedom in the mutant strain allowed it to eject the WT strain through the right trap boundary, just as in the ABM simulations. In particular, the rate of increase of the mutant strain fraction grew as the mutant strain fraction increased over *t* ∈ [1, 2], in accord with the positive feedback process observed in the ABM (see Fig 4C and S7 Movie). As in the ABM, we fit an exponential of the form *f*(*t*) = 0.5*e*^*α*(*t*−1)^ to the mean mutant strain fraction over *t* ∈ [1, 2] and found that the rate parameter *α* changed approximately linearly with *p*_rot,*o*_ (Fig 4C, inset, and S8 Fig). This linear dependence echoes the relation observed in the ABM simulations. Again, the similarity is evident when we plot *α* versus 1 − *p*_rot,o_ (compare Fig 3B and S9 Fig). This shows that the LM successfully captures the bulk forcing mechanism we observed in the ABM.

Unlike in the ABM, physical forces are not explicitly modeled in the LM. However, upon division a horizontal or vertical cell moves a column of cells consistent with its orientation. Hence, the average horizontal growth probability describes the propensity of one strain to displace the other in the horizontal direction. We therefore defined the mean horizontal growth propensity for cells of strain *k* as

$$\frac{1}{\sigma_{k}\left(t\right)}\sum_{i=1}^{M}\sum_{j=1}^{N}h^{\pm }\left(j\right)\delta_{k\gamma_{ij}},$$

where *σ*_*k*_(*t*) is the number of cells of strain *k* in the lattice at time *t*, *h*^±^ is the horizontal growth rate, *δ*_*ij*_ is the Kronecker delta function, and *γ*_*ij*_ is the strain identity of the cell at the *i*^th^ row and *j*^th^ column in the lattice. Fig 4D shows the temporal evolution of the average horizontal growth propensities of each strain for the second set of LM simulations (red curves). The average horizontal growth propensity of the mutant strain dominated that of the WT strain, explaining why the mutant strain ejected its competitor.

We observed one important difference in the single-interface simulations between the LM and the ABM. In the ABM simulations, the movement of the interface between the strains accelerated until the blue strain was completely ejected from the trap. By contrast, a deceleration phase followed the initial acceleration phase in the LM simulations. This difference was due to a less stable interface between the strains in the LM compared to the ABM. In particular, we observed that stray blue cells remained in the trap even after the majority of the blue population was ejected. Nevertheless, the LM captured both mechanisms of WT displacement observed in the ABM remarkably well, despite being considerably simpler, and more tractable (see S5 and S6 Figs). Indeed, the LM can be described exactly by a master equation (see S4 Fig) that can serve as a basis for further analysis.

### Collective signaling: A spatial consortial oscillator

As multi-strain collectives grow and fill microfluidic devices, spatial structure emerges: Single-strain bands form of various widths, which depend on the cell seeding, but large-width bands can exceed the diffusion correlation length of inter-strain communication [45]. We next suggest an experimentally feasible way to *control* such emergent spatial structures by using quorum-sensing (QS) signaling coupled to cell length modulation. In particular, we show computationally how oscillations in strain fraction emerge when a QS signal from one strain induces a reduction in average division length in the opposite strain in a negative feedback, dual-state switch topology.

#### ABM strain fraction oscillator

**Fig 5:**
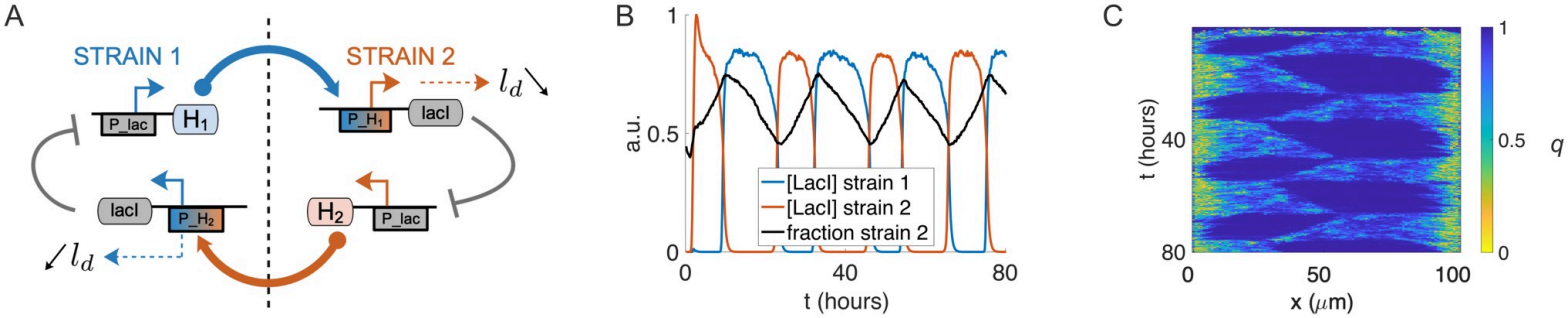
ABM oscillator. (A) Bi-stable circuit topology for quorum-sensing (QS) signal production, division length (*l*_*d*_) reduction, and QS repression by the protein LacI. (B) Sustained oscillations illustrated by the concentrations of LacI and strain fraction time series for an ABM simulation using the circuit topology in (A). Asymmetry in the strain fraction resulted from different QS molecules (C4HSL, C14HSL) with different diffusion rates. (C) Kymograph of order parameter *q* in the same simulation illustrating the switching behavior and emergent ordering dynamics (compare, Fig 3C).

We used our agent-based model (ABM) to suggest how spatiotemporal patterns in a microfluidic device can be controlled via changes in average division length. To do so we combined a *bistable* quorum sensing (QS) circuit based on the experimental circuit described in [12] with the division-length reduction circuit we used in our two-strain simulations (See Fig 5A). In this topology, each strain produces an orthogonal QS signal that activates the other strain’s LacI (a repressor protein) expression, which represses the production of QS signal in that strain. In our ABM simulations, when the QS signal received from the opposite strain surpassed a threshold concentration, *H*_*T*_, this also triggered the reduction in average cell division length. In simulations we reduced the division by a fixed factor, *a* = 0.6. Thus, in contrast to our previous simulations, average division length of a strain was effected by coupling to another strain rather than by *exogenous* induction. In the oscillator ABM simulations, each QS signal was well-mixed across the trapping region and coupled via the flow channels (see Methods).

We initialized these simulations by seeding the trap with 32 total cells, with each strain (chosen with equal probability) occupying separate halves of the trap. Due to the random initial seeding, upon trap-filling each strain occupied approximately one-half of the trap, as in the simulations shown in Fig 3. We used published diffusion rates (see S1 Appendix) for the orthogonal QS molecules C4HSL and C14HSL [12], and assigned C14HSL production to the orange strain and C4HSL to the blue strain.

In the oscillator topology, we call the non-induced strain the “long” strain (this is the strain whose signaling is not repressed by LacI). The QS signal produced by the long strain reaches the other strain to repress its QS production and induce its division length reduction (see Fig 5A). As expected from the simulations shown in Fig 3, we observed in ABM oscillator simulations that the short strain cooperatively ejected the long strain via the bulk forcing mechanism. As the fraction of the long strain in the trap decreased, the QS signal received by the short strain decreased and eventually fell below *H*_*T*_, turning off both division-length reduction and its QS signal repression by negative-feedback. At this point QS production in the previously induced (short) strain switched on, triggering division-length reduction and repression of QS signaling in the previously uninduced (long) strain. At this point the two strains exchanged places and began the next half-cycle of the oscillation.

This negative feedback topology produced sustained oscillations in the ABM (see S5 Movie). In Fig 5B, we plot the average LacI repressor concentration of each strain and the resulting strain fraction time series for an ABM simulation. A kymograph (Fig 5C) of the order parameter *q* (averaged as in Fig 3) shows the temporal transitions of cell ordering and resultant cooperative ejection of the majority strain in each oscillation half-period. We found that oscillations sustained indefinitely when the parameters were properly tuned, but with *H*_*T*_ set too low, extinction of a strain would result. To further understand the parameter dependence and to further explore the structure of the oscillator topology, we generated a reduced, differential equation model that focused on key parameters and separation of timescales, which we describe in the following section.

#### Reduced model of the consortial oscillator

To illuminate key features of the ABM consortial oscillator, we describe a fast-slow dynamical system that captures the ABM consortial oscillator dynamics and their dependence on various timescales. In this effective model, the dynamics manifest as a relaxation oscillation. The driver of the oscillation is a state variable, *Q*, which represents the division-length state of the microbial strains. We let *Q* = 1 represent the blue strain being in a reduced division length state, while 1 − *Q* = 1 (i.e., *Q* = 0) represents the same for the orange strain. Comparing with Fig 1A and 1B, *Q* is thus a proxy for the state of the QS promoter for LacI protein concentration in strain 1, which is ON concomitantly with reduced division length and thus determines the motion of the interface between the strains.

We simplified the trap geometry to one spatial dimension where the strain-interface front position is described by *x* ∈ [0, 1]. The dynamics for *Q* depend both on the proxy for concentration of blue strain (respectively, orange strain) QS signals, which we assume proportional to *x* (respectively, 1 − *x*), and to *Q* itself. In our reduced model we capture the QS sensitivity of each strain using ‘switching positions’: When *x* exceeds or falls below a specific value, denoted *K*_1_(*K*_2_), the blue strain (orange strain) switches division-length state through the action of an inverting Hill function, $L_{1}^{-}\left(L_{2}^{-}\right)$, which represents the QS production promoter (repressed by the protein LacI) in each strain. We thus capture the hysteresis of the toggle switch topology shown in Fig 5A with the bistability of the switch state, *Q*, in the region *x* between switching locations.

**Fig 6:**
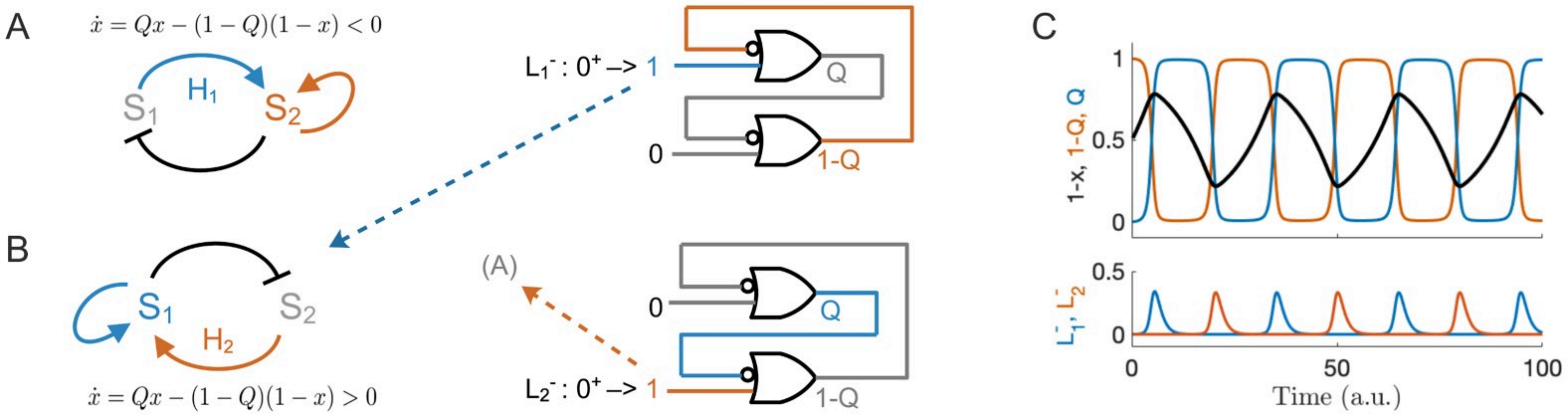
Consortial population relaxation oscillator. (A) Reduced oscillator model for *Q* = 0 (1 − *Q* = 1), which represents reduced cell length in the orange strain. In this state, the orange strain represses the blue strain by negative motion of the strain interface *x* ($\dot{x}<0$ represents ejection of the blue strain), which itself acts with positive feedback to increase the rate of motion. Signal *H*_1_ received from the blue strain activates this motion and will diminish by negative feedback (since the blue strain population is decreasing) eventually asserting the Hill function $L_{1}^{-}$, which will ‘set’ the memory state to *Q* = 1. (B) Complementary model for state *Q* = 1. Reduced cell length in the blue strain represses the orange strain by positive motion of the interface *x* ($\dot{x}>0$) eventually asserting the Hill function $L_{2}^{-}$ to ‘reset’ the state to *Q* = 0, in turn. (C) Top panel: Interface position 1 − *x* and complementary states 1 − *Q* (orange) and *Q* (blue) plotted vs. time for *K*_1_ = 0.2, *K*_2_ = 0.8, and *n* = 8 (see Eqs 2 and 3; cf. Fig 5B). Bottom panel: Hill functions $L_{1,2}^{-}$ fire when the interface position reaches the respective thresholds, thereby switching the state of the memory circuit. The relaxation oscillator requires separation of the switching and front-travel timescales (see main text).

Our reduced consortial oscillator model is

$$\dot{x}=Qx-(1-Q)(1-x)$$ (2)

$$\dot{Q}=\frac{1}{\epsilon }(-QL_{1}^{-}\left(x\right)+\left(1-Q\right)L_{2}^{-}\left(x\right)),$$ (3)

where $L_{1,2}^{-}$ are decreasing Hill functions of 1 − *x* and *x*, respectively. The Hill functions are defined with QS switching locations at *x* = *K*_1_, *K*_2_ ∈ [0, 1], such that

$$\begin{array}{c} L_{1}^{-}\left(x\right)≔\frac{1}{1+{\left(\frac{1-x}{1-K_{2}}\right)}^{n}}\;L_{2}^{-}\left(x\right)≔\frac{1}{1+{\left(\frac{x}{K_{1}}\right)}^{n}}, \end{array}$$ (4)

where *n* is the Hill exponent. The oscillations require *K*_1_ < *K*_2_ and *K*_2_ − *K*_1_ set to ensure separation of the timescales and sufficient switching time near the edges of the trap. Our reduced model assumes $\dot{x}$ dynamics are slow relative to *ϵ* (Eq 3), which models the physiological timescale to effect changes of aspect ratio once the front position, *x*, approaches either of the values *K*_1_, *K*_2_. In the limit of large *n*, and small *ϵ*, the interface velocity, $\dot{x}$, changes to positive (respectively, negative) as soon as *x* < *K*_1_ (*x* > *K*_2_). The values *K*_1,2_ thus define the two halves of the relaxation oscillation duty cycle. A simulation of Eqs 2 and 3 shows good agreement with our ABM result (Fig 6C), which supports the reduced-model as capturing the structure of the ABM oscillator of Fig 5.

## General discussion

Controlling the spatiotemporal population dynamics of distributed, microbial systems is essential for optimizing their functionality. Here we demonstrated how bacterial cell morphology can be used to control spatiotemporal strain dynamics in the close-packed, monolayer environment of a microfluidic trap.

**Fig 7:**
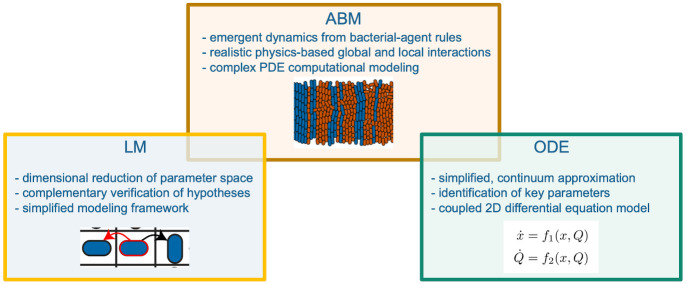
A schematic showing how the three models discussed here are related. The ABM (agent-based model) comprises a mechanical interaction physics-based model [48] coupled with a diffusion solver for realistic modeling of fast diffusion of intercellular QS signaling. Local rules for bacterial agents lead to emergent spatio-temporal dynamics as outlined in Results. The LM (lattice model) reduces modeling complexity by reducing the dimensionality of the parameter space, which simplifies explanations and testing of hypotheses. A continuum ODE (ordinary differential equation) model identifies key parameters of our oscillator model and abstracts the dynamics to a coupled 2D system with separated timescales.

We used both an agent-based model (ABM) and lattice model (LM) to show that reducing average cell division-length in a two-strain consortium confers a “mechanical fitness” advantage to the shorter-length strain. We also demonstrated theoretically how multi-strain consortia could alter strain fraction temporally using a quorum sensing network topology. Such strain fraction control in the ABM is described succinctly by a relaxation oscillator (Eqs 2–4 and Fig 6). We thus approached our problem by using different levels of granularity in the modeling and analysis. Fig 7 summarizes the relationship between the ABM, LM, and the model relaxation oscillator. Such multi-leveled analysis using different formalisms can help our understanding of biological systems whose dynamics evolve on multiple temporal and spatial scales [51].

Using our ABM, we showed strains in a consortium that achieved a competitive advantage via two distinct mechanisms that depended on the initial configuration of the strains. With a random seeding of strain types that resulted in mixed bands of each strain, we found that the emergence of horizontally-oriented cells can lead to invasion of adjacent columns of the opposite strain. In contrast, when two strains occupied different portions of the trap, the smaller strain was more disordered in a bulk population, which led to a lateral force that acted cooperatively to increase the number of disordered cells. Both strain configurations led to the ejection of the WT strain, by mechanical action of the smaller length mutant strain, to the microfluidic trap open boundaries.

The LM showed that both mechanisms can be explained by changes in a single parameter, the rotation probability, *p*_rot_. This parameter determined both the probability of invasion of a neighboring, opposite-strain column when the strains were intermingled, as well as the average lateral bulk force one strain exercised on the other, when the two strains occupied different parts of the trap.

Our ABM strain fraction oscillator and effective relaxation oscillation description showed that population control can be achieved using a negative-feedback, quorum-sensing signaling topology in an experimentally relevant genetic circuit [12], and we suggest that such a design could be extendable. With a small modification to our circuit, stable strain fractions could be *programmed* by adding exogenous inducer to, for example, repress the division length protein expression in one of the strains. Similarly, by removing the division-length circuit altogether in one of the strains, a maximum population size could result from single-ended control in some range of initial conditions. We suggest that desired strain fractions could be robustly generated under a wide range of initial population distributions. Such control of consortium composition is a fundamental problem in synthetic biology [10, 20], and our simulations suggest a method to relax dependence on necessary exogenous control [52, 53].

### Limitations

Are the mechanisms for controlling spatiotemporal dynamics of microbial communities biologically plausible? Zheng et al [43] controlled cell width and length independently by varying the expression levels of the cell division-regulatory proteins MreB and FtsZ, respectively. Moreover, there is numerical [42, 44] and experimental [54] evidence that cell shape affects spatial structures in crowded microbial colonies. However, we are not aware of examples of synthetic bacteria actively regulating their cell morphology in response to QS signals. Since genes that regulate cell length and width have been identified, and multicellular co-repressive circuits in which multiple strains communicate via QS signals have been built [12], we suggest the future construction of communities of synthetic organisms that regulate cell shape via quorum sensing signals. For simplicity, we have not included some factors that would impact the spatial organization of and inter-cellular communication within growing bacterial colonies in natural environments. For instance, we have ignored the mechanical interactions between cells and the extracellular matrix, which can impact the structures that emerge in biofilms [54, 55]. We have also not included the impact of fluid flow at the colony boundaries, which can affect the concentration of QS signals in a colony [56]. However, such factors can be controlled in synthetic communities of *E. coli* growing in microfluidic devices [37, 57, 58], creating conditions close to the ones in our models.

We made several assumptions to present the interactions within and across strains, and we explained the impact of these interactions in the simplest possible setting. However, these assumptions are not unreasonable: Our choice of the domain and boundary conditions was motivated by rectangular microfluidic traps used routinely by synthetic biologists [15]. The prevalent nematic ordering of cells in close-packed environments that is central to the emergence of spatio-temporal patterns has been observed experimentally [32, 37, 45, 59], and has been examined numerically [32, 48, 59] and analytically [32–34, 47, 48]. Although such nematic order is most likely to emerge in monolayer traps, it has been observed in colonies growing in three dimensions [54]. Thus, we can expect the type of close-packed interactions we described to occur—at least at the local level—between strains or even in lineages that differ in average division length.

Experiments have demonstrated that the total volume of two strains dividing at different average cell length can incrase at approximately the same rate [43]. However, in the case of strong nematic ordering in steady-state, differences in growth rates will not affect our results since the faster growing strain will just exit the trap at a higher rate in an aligned column (although initial conditions with different growth rates on trap-filling could significantly alter starting population fractions). If the cells are not strongly ordered (isotropic growth), we expect a significantly faster-growing strain would displace a slower growing strain due to differences in volume expansion rate. However, the range of relative growth rates valid for the interactions we have described here is yet to be determined.

## Conclusions

Understanding and controlling the behavior of distributed microbial systems is essential for engineering information exchange between constituent strains [21], ecological dynamics [22–24], metabolic resource allocation [25, 26], and microbial social interactions [27]. The active control of cell morphology provides a way to engineer the spatiotemporal dynamics in synthetic systems and to understand the patterns that emerge in microbial communities in nature.

## Methods

### Agent-based model

Our ABM captures mechanical interactions between cells growing in a microfluidic trap as well as the membrane and extracellular diffusion of signaling molecules produced intracellularly. We used this modeling framework to understand how changes in average cell length affect nematic cell ordering—and therefore emergent dynamics—in simulations of both homogeneous (single-strain) and competing (two-strain) microbial consortia. For two-strain simulations, we altered the mean division length of a strain with simulated external induction or by auto-regulatory control with quorum-sensing signaling topologies.

#### Cell growth, division, and induction

We modeled cell dynamics within a simulated, open-walled microfluidic device or *trap*. In ABM simulations, we assumed a monolayer rectangular trapping region with dimensions 20 × 100 *μ*m (height × width, see Figs 1 and 3). The trapping region is open on all sides to allow cell outflow. Two flow channels lie along the long edges of the device to provide nutrients and remove cells that exit the trapping region, thereby keeping the cell population approximately constant. Variants of this microfluidic design have been used in several published studies [10, 15, 45, 60]. We also used quorum-sensing communication between strains and in this case modeled the flow channels as well-mixed compartments of signaling concentration. That is, we ignored directional effects of the flow for simplicity [10, 15, 60]. Our ABM thus simulated a population of growing and dividing *E. coli* cells in a 2D microfluidic trap environment using a mechanical interaction algorithm we described previously [48]. Here, we extended our previous approach to include bacterial quorum-sensing (QS) communication by integrating the finite-element software *Fenics* [61–63] for numerical solution of the diffusion equation (see S1 Appendix for the complete ABM differential equations).

Cells were modeled as 2D spherocylinders of constant, 1 *μ*m width. Each cell grew exponentially in length with a doubling time of 20 minutes [10, 15]. In order to prevent division synchronization across the population, when a mother cell of length *l* divided, the two daughter cells were assigned random birth lengths *ϵ*_0_*l* and (1 − *ϵ*_0_)*l*, where *ϵ*_0_ was sampled independently at each division from a uniform distribution on [0.45, 0.55]. We set the division length of each daughter cell to $l_{d}={\left(2l_{0}\right)}^{1/2}{\left({\bar{l}}_{d}\right)}^{1/2}$, where *l*_0_ denotes the daughter’s birth length and ${\bar{l}}_{d}$ denotes the mean division length for the strain [64]. The mean division length, ${\bar{l}}_{d}$, was modulated in a strain in our ABM simulations by simulated external induction or by using a quorum-sensing communication network between two strains. We divided the intracellular proteins and signaling molecules in the mother cell between the two daughters in proportion to their birth length.

For two-strain simulations, we initialized the ABM in two different ways and colored the strains orange and blue for identification in simulation snapshots (see Figs 2 and 3, and S1 Appendix). We used in one case a spatially random initial seeding inside the trapping region (Fig 2 and S1 and S2 Movies). We chose the strain type of each seeded cell independently and with equal probability, which produced interchanging stripes of the two strains (Fig 2a) [45]. In another case we seeded the trapping region by placing blue cells in the left half and orange cells in the right half, which usually produced a single interface between the strains (Fig 3 and S3 and S4 Movies). In single-strain simulations, we used a neutral color for cell visualization (see Fig 1). Single-strain simulations were checked for strain fraction bias due to cell seeding with a negative control simulation.

To investigate how cell length affects emergent dynamics in two-strain consortia we reduced the mean division length (after a transient stabilization period for the population ratio) in the orange strain by a factor *a* ∈ [0.6, 0.85], so that the new mean division length satisfied ${\bar{l}}_{d,\text{new}}=a\times {\bar{l}}_{d,\text{original}}$ (the width of the cells is the same for both strains for the duration of the simulations, see Figs 2 and 3, and S1 and S4 Movies). Thus, the factor *a* determined the difference in cell length between the two strains.

#### Quorum-sensing communication

To model inter-strain communication and feedback topologies for strain fraction control, we extended our ABM to include diffusive intercellular signaling We coupled intercellular signaling to the ABM by using an ODE for membrane diffusion of QS signals: For each cell of a strain, the intracellular QS concentration, *H*, depended on production at rate *α* (which in general depends on the signal coupling from the other strain), first-order membrane diffusion kinetics at rate *d*, and the local external QS concentration, *H*_*e*_. Complete details are given in S1 Appendix.

We updated the external QS molecule concentrations over each time step by solving the 2D diffusion equation over the trapping region. To simulate the perimeter of the trapping region and storage of signaling molecules in the flow channels, we used a splitting method whereby signal flux from the trapping region was integrated into a homogeneous channel volume, and diluted at rate *γ* due to media flow [10, 15, 60, 65]. For two-strain consortia, we coupled signaling strength to cell-length control using a negative feedback topology. For strain 1, we reduced the mean division length of this strain when *H*_2_ > *H*_*T*_, where *H*_2_ is the measured intracellular QS signal from strain 2, and *H*_*T*_ is a threshold value (similarly for strain 2). Simulation code for our ABM and the integrated Fenics solver is open-source and available on Github (see S1 Appendix).

### Lattice model

To illuminate the mechanisms that drive patterning in bacterial collectives, we developed a lattice model (LM) that captures the essential features of cell growth and strain-strain interactions. Lattice models have a storied history in biological modeling and provide a valuable framework for modeling complex spatiotemporal dynamics in biological tissues [66–72]. As is typical of lattice models, our LM gains tractability at the expense of some fidelity to reality.

**Fig 8:**
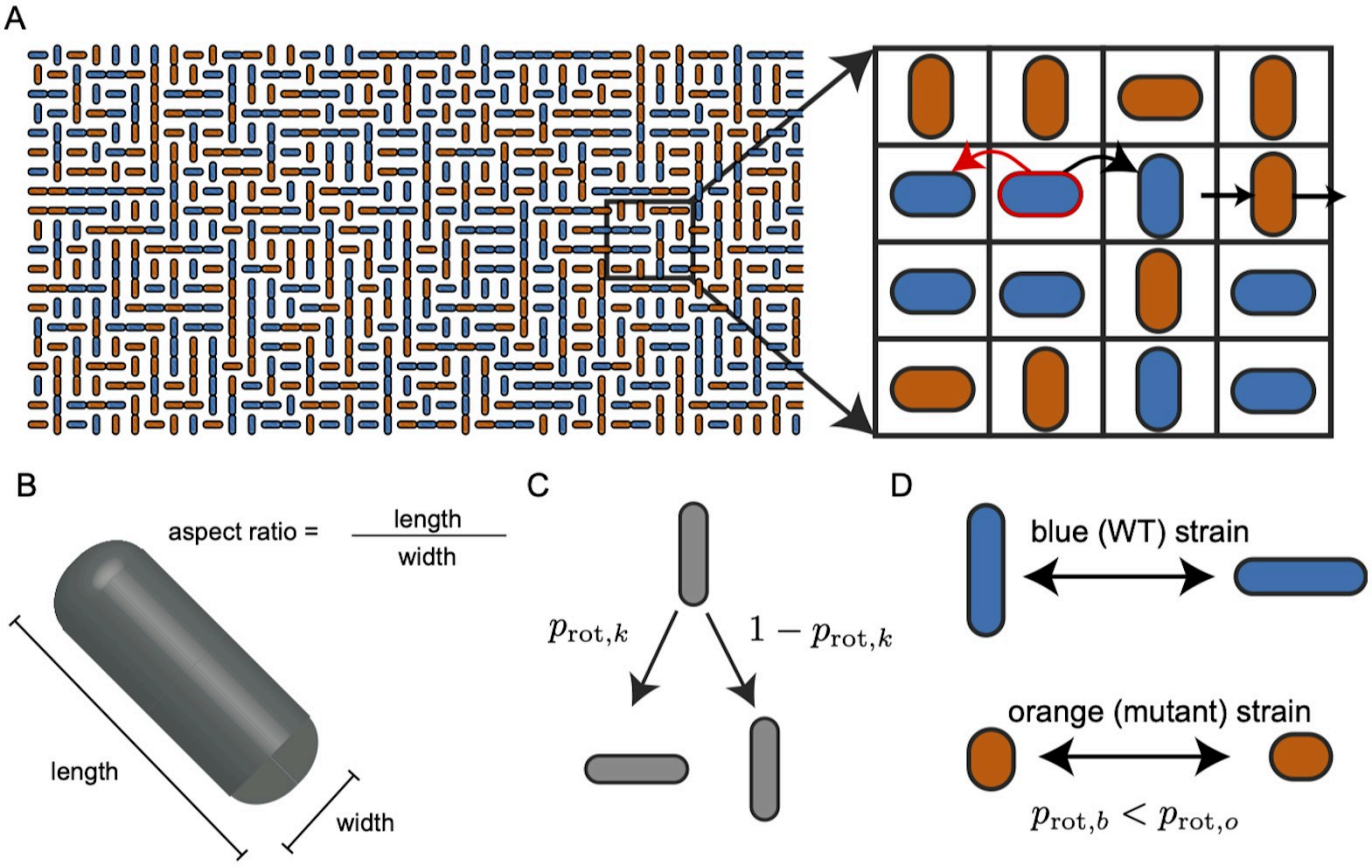
The lattice model (LM). **(A)** In the LM cell growth is directional and location-dependent: The horizontal cell outlined in red can grow to the right or left at location-dependent rates. The red arrow indicates that the leftward growth rate of the outlined cell is less than its rightward growth rate because this cell is located on the right side of the lattice. The black arrow indicates direction of cell division. When the cell divides, one daughter cell inherits the lattice position and orientation of the mother cell. The second daughter cell may rotate and occupies the lattice position immediately to the right of the mother cell, thereby displacing all existing cells in the direction of division by one unit. Cells that cross the lattice boundary are removed. **(B)** Schematic of a capsule-shaped bacterial cell. The cell’s aspect ratio is the ratio of its length to its width. **(C)** In two-strain LM simulations, the second daughter of a mother cell of strain type *k* rotates with probability *p*_rot,*k*_ if the 8 neighbors of the mother cell share her strain type. Otherwise, the second daughter rotates with a probability taken as the average of her probability of rotation and her 8 neighbors’ probabilities of rotation (see text for details). **(D)** We assume that a smaller aspect ratio corresponds to higher rotation probability in the LM to mimic the effect of a smaller division length in the ABM. We define *p*_rot,*o*_ as the probability that the daughter of an orange cell switches orientation upon birth, and define the equivalent parameter *p*_rot,*b*_ for cells of the blue strain similarly.

We modeled the rectangular microfluidic trap as an *M* × *N* lattice in $N^{2}$. In the model, locations in the lattice were occupied by vertically or horizontally oriented cells belonging to one of two strains. Cells grew at location-dependent rates, as defined below. Upon division, one of the daughter cells replaced the mother cell, while the second daughter cell displaced a neighbor and thus moved every cell in the direction of growth by one lattice site (see Fig 8a). We modeled traps with no walls (i.e. absorbing boundaries), so any cell that crossed the boundary of the lattice disappeared from the system. Times between divisions in the trap were independent of one another and exponentially distributed, with mean determined by the sum of growth rates of the cells in the trap. The locations of the division events were also independent of one another.

The assumption that cell division displaces the entire half-column or half-row in the direction of growth is strong. The impact of cell division may result in only local displacement [32, 34, 47, 48]. Nevertheless, we found that changing how many cells are moved after a cell division does not significantly alter qualitative behavior in our LM simulations.

We denote by *v*^±^(*i*) the growth rate of a vertical cell in the *i*^th^ row toward the top (+) or bottom (–) boundary and by *h*^±^(*j*) the growth rate of a horizontal cell in the *j*^th^ column toward the right (+) or left (–) boundary. We assumed that growth rates were modulated by the population that lies between a given cell and the closest boundary in the direction of growth, and hence that the growth rate of a vertical (horizontal) cell depended only on the row (column) in which it resided. We used a single parameter *κ* ∈ [0, ∞) to characterize how strongly the population modulated growth and set

$$\begin{array}{c} v^{+}\left(i\right)=\lambda e^{\left(-\kappa (M-i)\right)}\;v^{-}\left(i\right)=\lambda e^{\left(-\kappa (i-1)\right)} \end{array}$$ (5a)

$$\begin{array}{c} h^{+}\left(j\right)=\lambda e^{\left(-\kappa (N-j)\right)}\;h^{-}\left(j\right)=\lambda e^{\left(-\kappa (j-1)\right)}. \end{array}$$ (5b)

Using different decaying functions for *v*^±^ and *h*^±^ did not alter LM dynamics significantly [33]. Since the growth rate determines the rate at which a cell divides we use the two terms interchangeably.

#### Cell division and rotation

Previous simulations and experiments have shown that in crowded environments cell orientations evolve dynamically due to interactions with neighbors [32–34, 37, 47]. Rotational freedom increases as characteristic cell lengths decrease [37, 44]. Our ABM simulations also showed that nematic disorder grows as mean cell division length decreases (see Fig 1). Since cells in the LM do not have physical dimensions, we introduced rotation probabilities, *p*_rot,*k*_, that determined the likelihood that cells in strain *k* change orientation from vertical to horizontal or vice versa. Therefore, based on our ABM results, and previous experimental observations, we assumed that the probability of rotation increased monotonically with a decrease in mean cell division length. The precise dependence of *p*_rot,*k*_ on cell morphology is complicated. For simplicity we assumed that the intrinsic rotation probability of a cell is 1 − *p*_rot,*k*_ = *a*, where *a* is the normalized average division length of the smaller strain. As we discuss below, this probability can be affected by a cell’s neighbors.

In our simulations, a cell rotation only occurred at the moment of cell division. When a mother cell divided, the daughter cell that occupied the lattice site vacated by the mother did not rotate. The orientation of the daughter cell that displaced a neighbor differed from that of the mother cell with probability *p*_rot,*k*_ when the mother cell and her eight neighbors shared the same strain type (see Fig 8C). In crowded environments, the rotation dynamics of a given cell depends on the morphology of surrounding cells: If the surrounding cells are less likely to rotate, and hence better aligned, the given cell will be less likely to rotate as well. To account for this, in two-strain LM simulations, we set the displacing daughter’s rotation probability to the average rotation probability of itself and its eight neighbors. We implemented this model using a Gillespie algorithm [73] with the following events and corresponding rates: A a vertical (horizontal) cell of strain *k* at location (*i*, *j*) in the lattice displaced a neighbor at location (*i* ± 1, *j*) (respectively (*i*, *j* ± 1)) by producing a copy of equal orientation with probability *v*^±^(*i*)(1 − *p*_rot,*k*_)Δ*t* (respectively *h*^±^(*j*)(1 − *p*_rot,*k*_)Δ*t*) and of differing orientation with probability *v*^±^(*i*)*p*_rot,*k*_Δ*t* (respectively *h*^±^(*j*)*p*_rot,*k*_Δ*t*). Our LM results coincided remarkably well with our ABM results (see Results and Discussion). As with ABM simulations, we assigned a unique color to each strain for visualization.

## Supporting information

S1 Appendix Supporting information file. Text sections on code availability, model descriptions and parameter tables, and a glossary.
(PDF)

S1 Movie ABM invasion mechanism *a* = 0.6. Example ABM simulation movie file with *a* = 0.6 and random strain cell-seeding corresponding to Fig 2 in the main text.
(MP4)

S2 Movie ABM invasion mechanism *a* = 0.7. Example ABM simulation movie file with *a* = 0.7 and random strain cell-seeding corresponding to Fig 2 in the main text.
(MP4)

S3 Movie ABM pushing mechanism *a* = 0.6. Example ABM simulation movie file with *a* = 0.6 and separated strain cell-seeding corresponding to Fig 3 in the main text.
(MP4)

S4 Movie ABM pushing mechanism *a* = 0.7. Example ABM simulation movie file with *a* = 0.7 and separated strain cell-seeding corresponding to Fig 3 in the main text.
(MP4)

S5 Movie ABM oscillator. Example ABM simulation movie file of the consortial oscillator. The reduced cell length by factor *a* = 0.6 switches in a bi-stable signaling circuit.
(MP4)

S6 Movie Invasion in the LM. Video demonstration of invasion in the LM. When the equilibrium stripe distribution is sporadic, the strain that is more likely to rotate (orange) invades the blue stripes and flushes them out.
(MP4)

S7 Movie Bulk forcing in the LM. Video demonstration of bulk forcing in the LM. When the equilibrium stripe distribution consists of a single interface, the cells of the smaller strain (orange) cooperatively flush out the blue cells.
(MP4)

S1 Fig ABM time series for random cell seeding. Simulation time series for the ABM for various aspect ratios, *a* (see main text Fig 2). A-F correspond to *a* = 0.6, 0.65, 0.7, 0.75, 0.8, 0.85. Values for *α* are in units [min^−1^] and were computed using a least-squares fit to 0.5(1 − *e*^−*αt*^) after the start of induction (vertical thin blue line) and up to the strain ratio of 0.95.
(TIF)

S2 Fig ABM time series for partitioned cell seeding. Simulation time series for the ABM for various aspect ratios, *a* (see main text Fig 3). A-F correspond to *a* = 0.6, 0.65, 0.7, 0.75, 0.8, 0.85. Values for *α* are in units [min^−1^] and were computed using a least-squares fit to 0.5*e*^*αt*^ after the start of induction (vertical thin blue line) and up to the strain ratio of 0.95.
(TIF)

S3 Fig ABM invasion mechanism for different aspect ratios. Each panel A,B is a sequence of 3 ABM snapshot frames moving forward in time. After reduction of the average division length in the orange strain, we observed the appearance of horizontally-oriented cells that invaded adjacent columns. In some cases (but not all, compare left and right examples), invasion resulted in occupation of the mother-cell position of the column, which altered the strain fraction by subsequent ejection of the previous strain to the top/bottom open trap boundaries. Decreased frequency of invasion events correlated with increasing *a* (see main text). Column A: simulation snapshots for cell length reduction factor *a* = 0.6. Circle in middle panel indicates observance of horizontally oriented orange cell in the bulk. Green arrow indicates a flow of time after this observation to circle in the bottom panel, which shows a resulting local change in the strain fraction (a blue column was invaded and replaced by the orange strain). Column B: same for *a* = 0.7. Circle in middle panel again indicates observance of horizontally oriented orange cell in the bulk. Similarly, green arrow indicates a flow of time, but here the invasion did not succeed, resulting in no change of local strain fraction.
(TIF)

S4 Fig Master equation solutions. Solutions to the master equation, Text D in S1 Appendix, for different initial conditions. (a) When the initial condition consists of two stripes–each one consisting of a single strain of cells, the solution manifests as a traveling front. (b) When initial data consists of stripes of more sporadic width and spatial location, the dynamics resemble the invasion mechanisms described in the main text.
(TIF)

S5 Fig Mechanisms in the LM. Sample simulations of the LM with different initial conditions illustrating the bulk force and invasion mechanisms.
(TIF)

S6 Fig Invasion details in LM. Fine-grained demonstration of how invasion occurs in the LM.
(TIF)

S7 Fig Dynamics of LM. In the LM, a population of cells with a given *p*_rot_ value will reach an equilibrium value for the fraction of cells that are horizontal. For these simulations, we begin all cells vertically. We find that if *p*_rot_ is sufficiently large, then about half the population will be horizontal at any given time.
(TIF)

S8 Fig Noise in LM fits. Further illustrations of invasion and bulk forcing in the LM. Top row: bulk forcing. Bottom row: invasion. For distinct *p*_rot_ values, we average several trajectories to obtain an average strain fraction temporal dynamics. We then fit exponential curves to the average time series to obtain the results seen in the insets of Fig 4 of the main text. For the top row, we fit the average dynamics to a function of the form 0.5*e*^*αt*^. For the bottom row, we fit the average dynamics to a function of the form 0.5(1 − *e*^−*αt*^). The resulting *α* versus 1 − *p*_rot_ relations can be seen in the insets of Fig 4.
(TIF)

S9 Fig Lattice model simulations. A replica of Fig 4 from the main text, but with *q* and *α* now plotted against 1 − *p*_rot_. The trends here more directly mirror the trends shown in the ABM.
(TIF)
