# Supplementary material for: Emergent spatiotemporal population dynamics with cell-length control of synthetic microbial consortia
Source: PLoS Comput Biol. 2021 Sep 22;17(9):e1009381. doi: 10.1371/journal.pcbi.1009381 (PMC8489724; doi:10.1371/journal.pcbi.1009381)
Supplement: S1 Appendix — Text sections on code availability, model descriptions and parameter tables, and a glossary. (PDF) [file pcbi.1009381.s001.pdf]

SUPPORTING INFORMATION: APPENDIX

A. Data Availability

Simulation C++ code for the ABM can be found here: https://github.com/jwinkle/eQ. ABM simulations can be run natively on Linux (e.g. laptop or cluster computing enviornments) with proper (but cumbersome) installation of Fenics but can be more simply run with installation of Docker on MacOS, Linux or Windows environments. Details of the current installation procedures to install Fenics within Docker are given here: https://fenics.readthedocs.io/en/latest/installation.html#containers-docker-linux-macos-and-windows-64-bit and are subject to change in future Fenics updates. See https://fenicsproject.org/download/ for other installation pathways. ABM installation script instructions within a Docker environment can be found in the README of the above github repository, and code used for simulation results can be found under the PLoSCompBio21 branch.
All code used to produce figures in the main and supplemental document pertaining to the lattice model (LM) are available at https://github.com/Bargo727/AspectRatio. Illustrative videos for the LM are also available in the same repository.

B. ABM Oscillator Algorithm

The ABM oscillator was modeled using the following differential equations for the intracellular concentrations of the two HSL molecules, H_1 and H_2, (both of which are present in each strain) and LacI concentration, $L_{1,2}$ (only for each strain 1,2, respectively). For $i \in \{L, 1, 2\}$, α_i is the respective protein or HSL production rate. The parameters $n_{H,L}$ are the Hill exponents and $K_{H,L}$ are the HSL (respectively LacI) concentrations for which the governed production rate is half-maximum. The dilution rate $\gamma_d = \log 2/\tau_d$ (where τ_d is the cell doubling time) models the concentration dilution due to cell growth. The diffusion rate for HSL across the cell membrane is given by $d_{1,2}$ for each HSL and for $i \in \{1, 2\}, H_{i,e}$ is the local external HSL concentration from the Fenics model (see below). Our model is based on that given in Kim et al., 2019 (see main text references). Numerical values for the parameters used in the simulations are given in Table 1, below.
The ODE for strain 1 are

$$\dot{L}_1 = \alpha_L \frac{(\frac{H_2}{K_H})^{n_H}}{1 + (\frac{H_2}{K_H})^{n_H}} - \gamma_d L_1 \tag{B.1}$$

$$\dot{H}_1 = \alpha_1 \frac{1}{1 + (\frac{L_1}{K_L})^{n_L}} - d_1(H_1 - H_{1,\mathrm{e}}) - \gamma_d H_1 \tag{B.2}$$

$$\dot{H}_2 = -d_2(H_2 - H_{2,\mathrm{e}}) - \gamma_d H_2 \tag{B.3}$$

The corresponding, equations for each cell in strain 2 are

$$\dot{L}_2 = \alpha_L \frac{(\frac{H_1}{K_H})^{n_H}}{1 + (\frac{H_1}{K_H})^{n_H}} - \gamma_d L_2 \tag{B.4}$$

$$\dot{H}_2 = \alpha_2 \frac{1}{1 + (\frac{L_2}{K_L})^{n_L}} - d_2(H_2 - H_{2,\mathrm{e}}) - \gamma_d H_2 \tag{B.5}$$

$$\dot{H}_1 = -d_1(H_1 - H_{1,\mathrm{e}}) - \gamma_d H_1 \tag{B.6}$$

The diffusion of external HSL concentrations, $H_{1,\mathrm{e}}, H_{2,\mathrm{e}}$, in the microfluidic trap space was modeled using diffusion coefficents $D_{1,2}$. The external HSL equations are

$$\dot{H}_{1,\mathrm{e}} = D_1 \nabla^2 H_1 + d_1(H1 - H_{1,\mathrm{e}}) \tag{B.7}$$

$$\dot{H}_{2,\mathrm{e}} = D_2 \nabla^2 H_2 + d_2(H2 - H_{2,\mathrm{e}}) \tag{B.8}$$

subject to boundary conditions $H_{i,\mathrm{e}}(x,t) = H_{i,\mathrm{c}}(x,t)$ for $i \in \{1,2\}$, $x \in \Gamma$, where Γ is the trap boundary. We solved these equations using the finite-element software Fenics (see fenicsproject.org and main text references). Here $H_{1,\mathrm{c}}$, and $H_{2,\mathrm{c}}$ are the concentrations of the two HSL molecules in the channel that surrounds the microfluidic trap. We used a compartment model that computed the flux into the channel $J_{i,\mathrm{c}} = -D_i \nabla H_{i,\mathrm{e}}$, for each HSL, $i \in \{1,2\}$, over the boundary Γ, and diluted this concentration due to media flow at velocity v_c according to

$$\dot{H}_{1,\mathrm{c}} = D_1 \nabla H_{1,e} \cdot dS - \gamma_c H_{1,\mathrm{c}} \tag{B.9}$$

$$\dot{H}_{2,\mathrm{c}} = D_2 \nabla H_{2,e} \cdot dS - \gamma_c H_{2,\mathrm{c}} \tag{B.10}$$

where dS is the area of the trap/channel boundary, and $\gamma_c := v_c/l_c$, where l_c is the length of the trap/channel boundary.

C. Parameter Table for the ABM oscillator

**ABM Oscillator Parameter Table**	
γ_d	$\log 2/20$ [min^{-1}]
α_L	γ_d
α_1	2300 [min^{-1}]
α_2	1500 [min^{-1}]
K_H	0.35
n_H	8
K_L	0.5
n_L	8
d_1 (C4HSL)	3 [min^{-1}]
d_2 (C14HSL)	2.1 [min^{-1}]
D_1 (C4HSL)	3e4 [μm^2 min^{-1}]
D_2 (C14HSL)	2.1e4 [μm^2 min^{-1}]
γ_c	0.1 [min^{-1}]

The value for γ_d is computed for a 20 minute cell doubling time in the ABM. All concentrations are unitless, normalized to 1 for the peak intracellular concentration of HSL for each molecule. Values for HSL diffusion rates are from Pai and You, 2009 (see main text references).

D. Lattice Model Master Equation

To understand the dynamics of the lattice model we develop a master equation describing the time evolution of occupation probabilities at different sites. Let $p_{ij}^{c,k}$ denote the probability that a cell at site ij is of strain $k = 1, 2$ and has orientation $c = v, h$. The dynamical equations for these probabilities on the lattice involve two– and three–point correlations and are not helpful even if written down in full. We can close the resulting equation at the first moment and obtain

$$
\begin{aligned}
\frac{dp_{ij}^{v,k}}{dt} = \Big(& v_\kappa^+(i-1)p_{(i-1)j}^{v,k}(1-p_{rot,k}) + p_{(i-1)j}^{v,k} \sum_{\alpha=\max\{1,i-1-R\}}^{i-2} (v_\kappa^+(\alpha)(p_{\alpha j}^{v,k} + p_{\alpha j}^{v,\neg k})) \\
& + v_\kappa^-(i+1)p_{(i+1)j}^{v,k}(1-p_{rot,k}) + p_{(i+1)j}^{v,k} \sum_{\alpha=i+2}^{\min\{M,i+1+R\}} (v_\kappa^-(\alpha)(p_{\alpha j}^{v,k} + p_{\alpha j}^{v,\neg k})) \\
& + p_{i(j-1)}^{v,k} \sum_{\alpha=\max\{1,j-1-R\}}^{j-2} (h_\kappa^+(\alpha)(p_{i\alpha}^{h,k} + p_{i\alpha}^{h,\neg k})) + p_{i(j+1)}^{v,k} \sum_{\alpha=j+2}^{\min\{N,j+1+R\}} (h_\kappa^-(\alpha)(p_{i\alpha}^{h,k} + p_{i\alpha}^{h,\neg k})) \\
& + h_\kappa^+(j-1)p_{i(j-1)}^{h,k}p_{rot,k} + h_\kappa^-(j+1)p_{i(j+1)}^{h,k}p_{rot,k}\Big) \times (1-p_{ij}^{v,k}) \\
& - \Big((p_{(i-1)j}^{h,\neg k} + p_{(i-1)j}^{h,k} + p_{(i-1)j}^{v,\neg k}) \sum_{\alpha=\max\{1,i-1-R\}}^{i-2} (v_\kappa^+(\alpha)(p_{\alpha j}^{v,k} + p_{\alpha j}^{v,\neg k})) \\
& \quad + v_\kappa^+(i-1)p_{(i-1)j}^{v,\neg k} + v_\kappa^+(i-1)p_{(i-1)j}^{v,k}p_{rot,k} \\
& \quad + (p_{(i+1)j}^{h,\neg k} + p_{(i+1)j}^{h,k} + p_{(i+1)j}^{v,\neg k}) \sum_{\alpha=i+2}^{\min\{M,i+1+R\}} (v_\kappa^-(\alpha)(p_{\alpha j}^{v,k} + p_{\alpha j}^{v,\neg k})) \\
& \quad + v_\kappa^-(i+1)p_{(i+1)j}^{v,\neg k} + v_\kappa^-(i+1)p_{(i+1)j}^{v,k}p_{rot,k} \\
& \quad + (p_{i(j-1)}^{h,\neg k} + p_{i(j-1)}^{h,k} + p_{i(j-1)}^{v,\neg k}) \sum_{\alpha=\max\{1,j-1-R\}}^{j-2} (h_\kappa^+(\alpha)(p_{i\alpha}^{h,k} + p_{i\alpha}^{h,\neg k})) \\
& \quad + h_\kappa^+(j-1)p_{i(j-1)}^{h,\neg k} + h_\kappa^+(j-1)p_{i(j-1)}^{h,k}(1-p_{rot,k}) \\
& \quad + (p_{i(j+1)}^{h,\neg k} + p_{i(j+1)}^{h,k} + p_{i(j+1)}^{v,\neg k}) \sum_{\alpha=j+2}^{\min\{N,j+1+R\}} (h_\kappa^-(\alpha)(p_{i\alpha}^{h,k} + p_{i\alpha}^{h,\neg k})) \\
& \quad + h_\kappa^-(j+1)p_{i(j+1)}^{h,\neg k} + h_\kappa^+(j+1)p_{i(j+1)}^{h,k}(1-p_{rot,k})\Big) \times p_{ij}^{v,k}
\end{aligned}
\tag{D.1}
$$

Though complicated, Eq. (D.1) is able to capture both mechanisms: bulk displacement and invasion (see Fig 4). We can see that the Eq. (D.1) captures the two mechanisms described in the main text from the LM well (compare S4 Fig to S5 Fig). Unfortunately, further analysis is not possible with this model.

E. Glossary

constituent strains—the component bacterial strains that form a microbial consortium. Also: **constituent parts**.

distributed microbial system—a type of microbial consortium wherein distinct microbial strains contain distinct parts of a gene circuit so that they collectively perform some logical function.

fitness—the ability of a microbial strain to reproduce successfully in a given environment (e.g. a 2D microfluidic environment).

growth-expansion force—the force exerted by a bacterial cell on its surroundings (and vice-versa) due to its axial growth and due to barriers and axial growth of all other cells in its environment.

mechanical fitness—fitness due to physical properties of a cell, including through mechanical interactions with its environment.

microbial consortium—a collection of two or more genetically distinct bacterial species/strains. Also: **bacterial consortia**, **bacterial collective**, **microbial collective**, **multi-strain collective**

microbial strain—referring to a population of genetically identical bacteria, typically a part of a microbial consortium. Also: **strain**, **bacterial strain**.

mother-cell position—in columnar bands formed by rod-shaped bacteria in open-walled, 2D spatially extended microfluidic traps, the approximate symmetric center of the column where a mother-cell divides to produce all progeny of the column in both directions.

nematic ordering—a term borrowed from liquid-crystal modeling, here a measure of non-polar ordering of rod-shaped bacteria. Also: **cell ordering**, **nematic cell ordering**, **ordering**.

orthogonal quorum sensing signals—quorum sensing molecules whose signal-receptor pairs have no or limited cross-talk and effectively operate independently of each other.

strain fraction—in microbial consortia, a measure of the population of a given strain relative to the entire population of a consortium, typically measured by cell number or volume occupied. Also: **strain ratio**, **population ratio**

synthetic gene circuit—a genetically engineered assembly of biological parts encoding RNA or protein that enables target cells to perform some logical function.

synthetic microbial consortia—microbial consortia with a synthetic gene circuit forming a distributed microbial system, whose functionality is distributed over strains (typically 2 to 3), usually to improve robustness or introduce spatial behavior. Also: **synthetic bacterial collective**
