# Supplementary figures and images for: Emergent spatiotemporal population dynamics with cell-length control of synthetic microbial consortia
Source: PLoS Comput Biol. 2021 Sep 22;17(9):e1009381. doi: 10.1371/journal.pcbi.1009381 (PMC8489724; doi:10.1371/journal.pcbi.1009381)

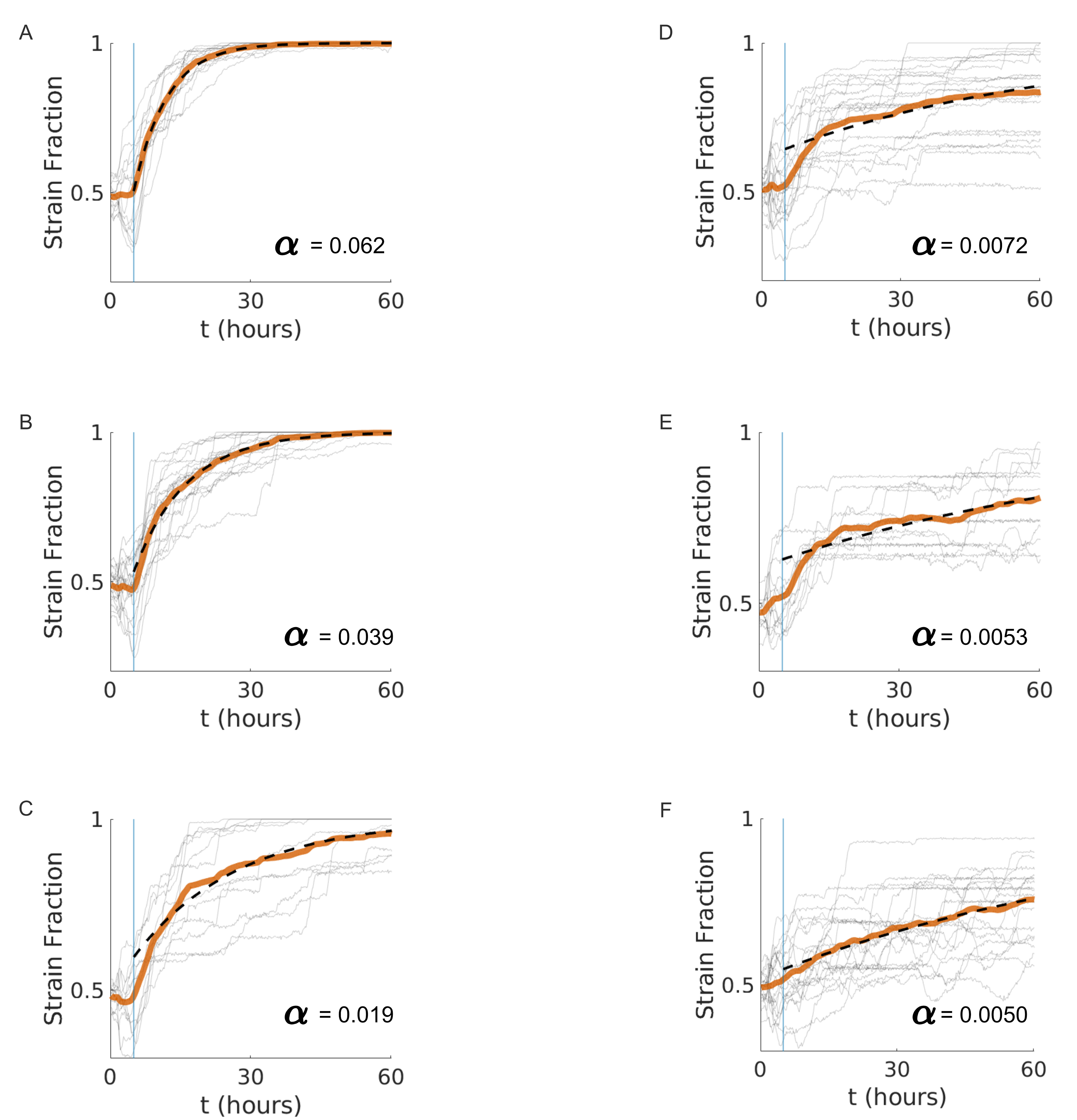

Supplement: S1 Fig — Simulation time series for the ABM for various aspect ratios, a (see main text Fig 2). A-F correspond to a = 0.6, 0.65, 0.7, 0.75, 0.8, 0.85. Values for α are in units [min−1] and were computed using a least-squares fit to 0.5(1 − e−αt) after the start of induction (vertical thin blue line) and up to the strain ratio of 0.95. (TIF) [file pcbi.1009381.s009.tif]

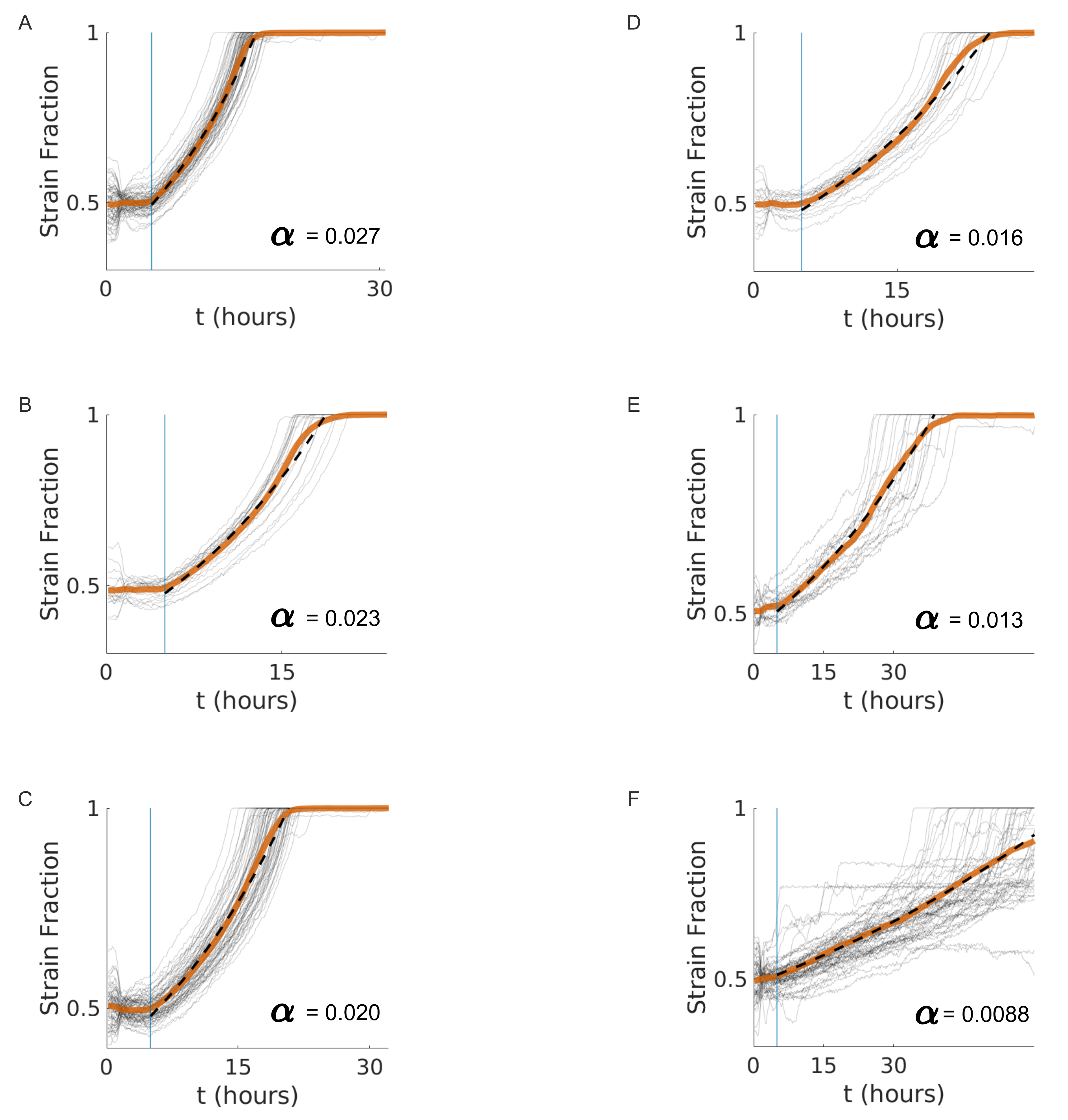

Supplement: S2 Fig — Simulation time series for the ABM for various aspect ratios, a (see main text Fig 3). A-F correspond to a = 0.6, 0.65, 0.7, 0.75, 0.8, 0.85. Values for α are in units [min−1] and were computed using a least-squares fit to 0.5eαt after the start of induction (vertical thin blue line) and up to the strain ratio of 0.95. (TIF) [file pcbi.1009381.s010.tif]

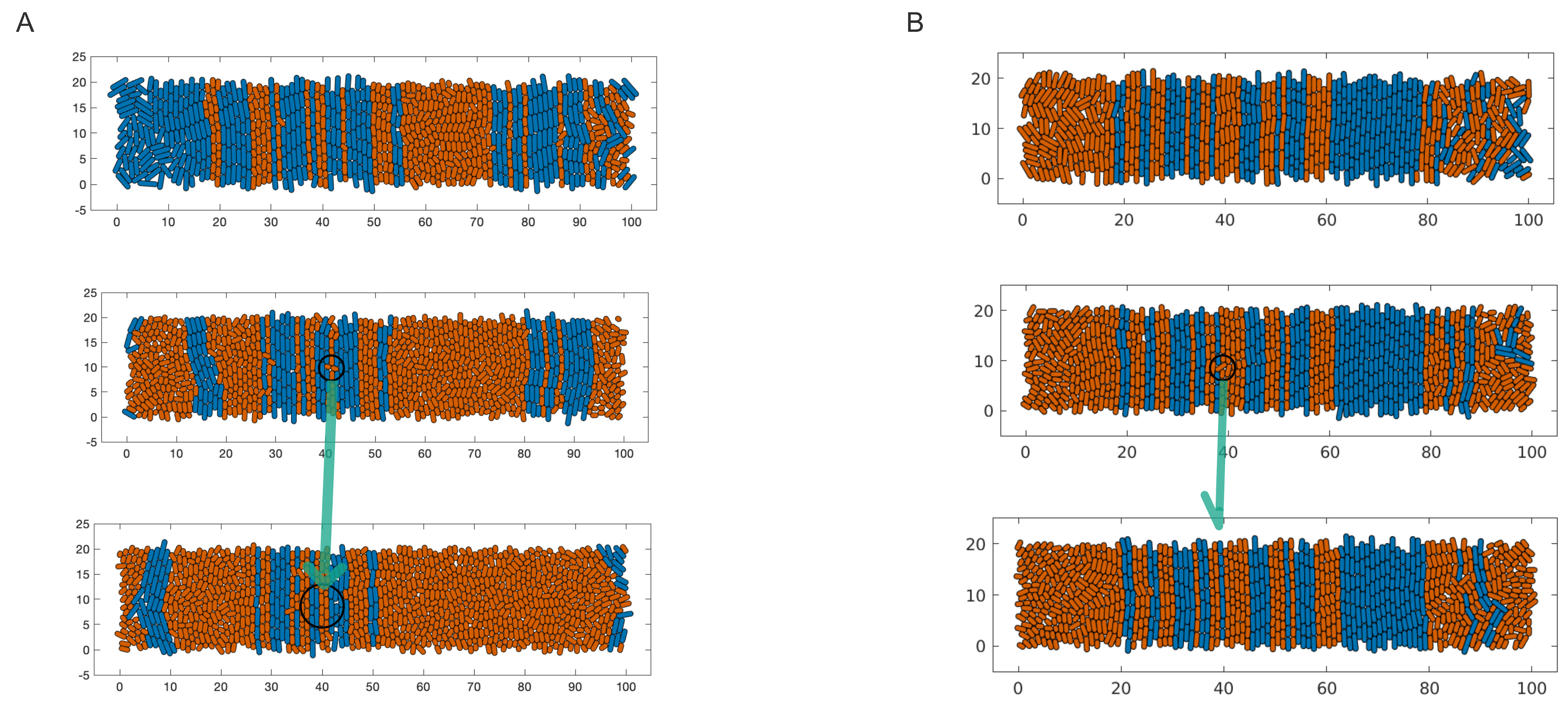

Supplement: S3 Fig — Each panel A,B is a sequence of 3 ABM snapshot frames moving forward in time. After reduction of the average division length in the orange strain, we observed the appearance of horizontally-oriented cells that invaded adjacent columns. In some cases (but not all, compare left and right examples), invasion resulted in occupation of the mother-cell position of the column, which altered the strain fraction by subsequent ejection of the previous strain to the top/bottom open trap boundaries. Decreased frequency of invasion events correlated with increasing a (see main text). Column A: simulation snapshots for cell length reduction factor a = 0.6. Circle in middle panel indicates observance of horizontally oriented orange cell in the bulk. Green arrow indicates a flow of time after this observation to circle in the bottom panel, which shows a resulting local change in the strain fraction (a blue column was invaded and replaced by the orange strain). Column B: same for a = 0.7. Circle in middle panel again indicates observance of horizontally oriented orange cell in the bulk. Similarly, green arrow indicates a flow of time, but here the invasion did not succeed, resulting in no change of local strain fraction. (TIF) [file pcbi.1009381.s011.tif]

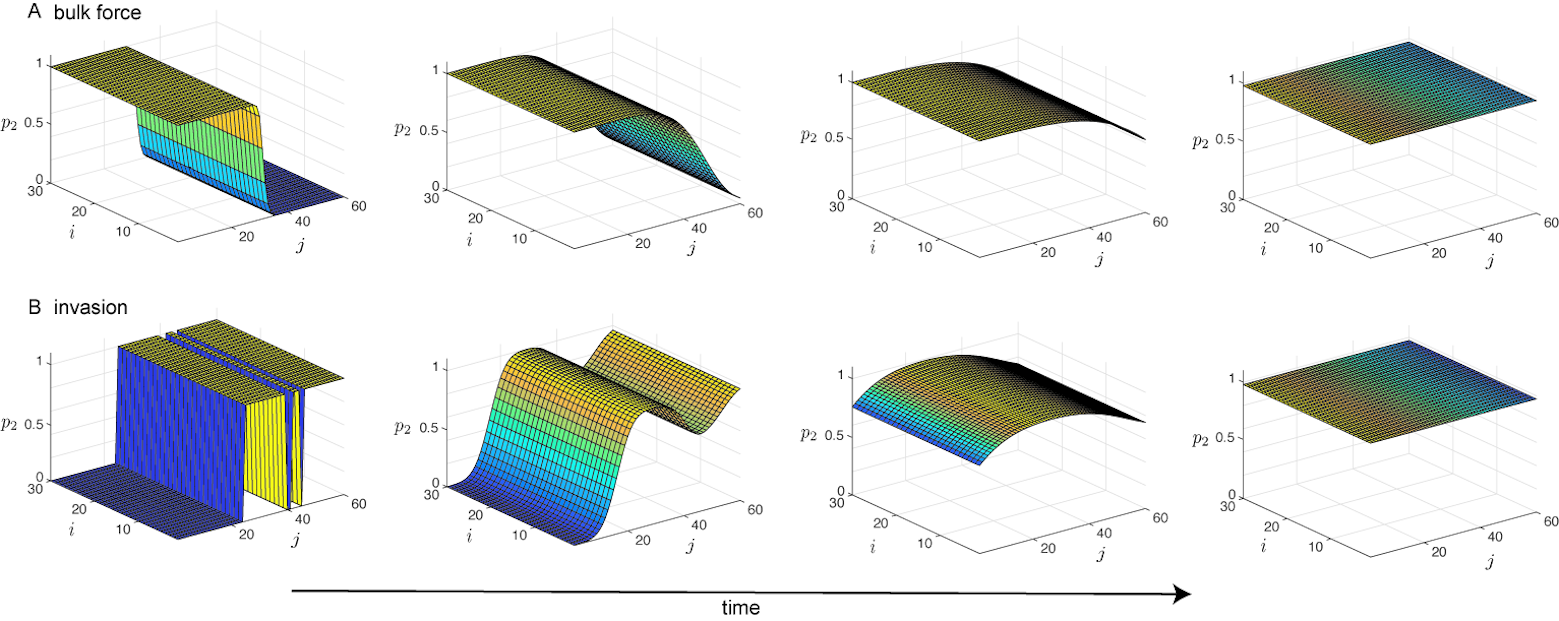

Supplement: S4 Fig — Solutions to the master equation, Text D in S1 Appendix, for different initial conditions. (a) When the initial condition consists of two stripes–each one consisting of a single strain of cells, the solution manifests as a traveling front. (b) When initial data consists of stripes of more sporadic width and spatial location, the dynamics resemble the invasion mechanisms described in the main text. (TIF) [file pcbi.1009381.s012.tif]

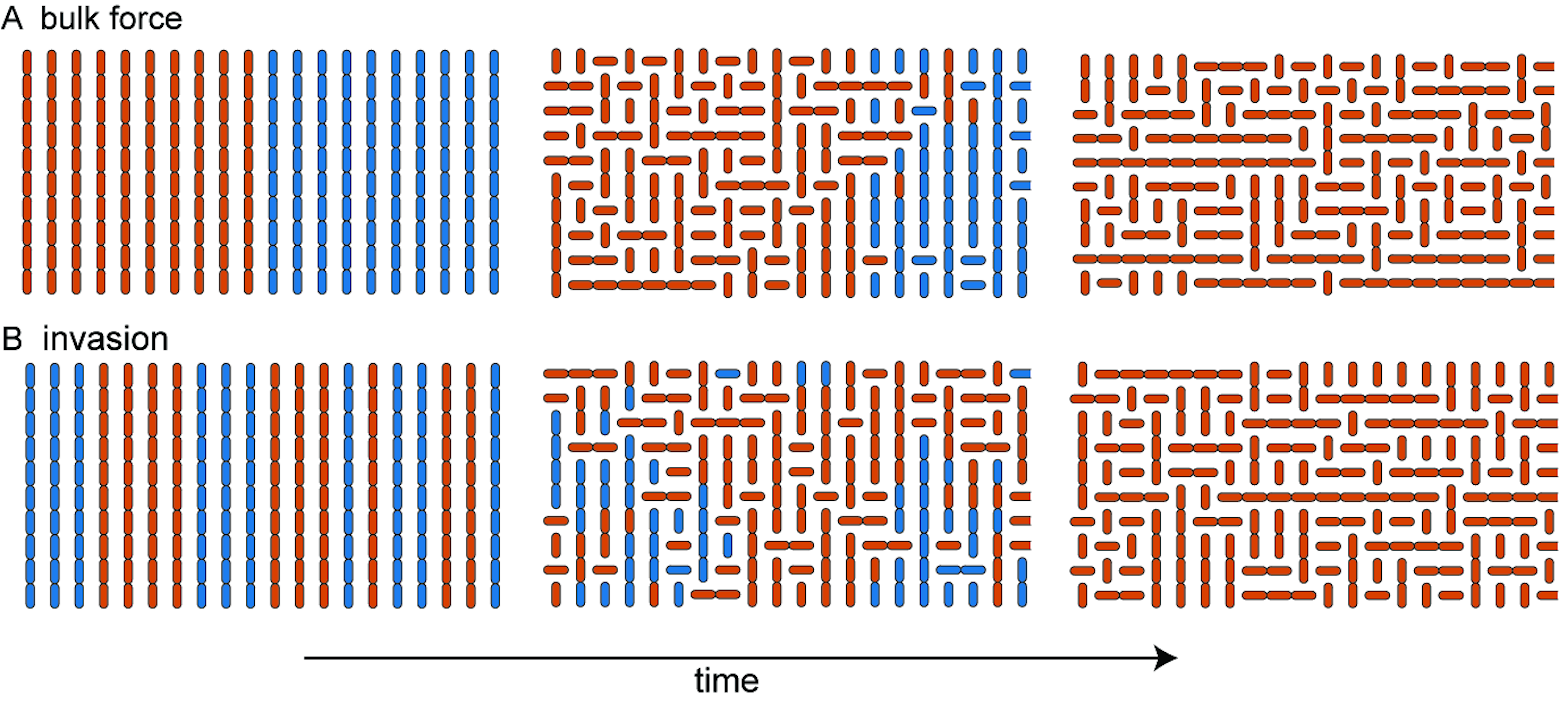

Supplement: S5 Fig — Sample simulations of the LM with different initial conditions illustrating the bulk force and invasion mechanisms. (TIF) [file pcbi.1009381.s013.tif]

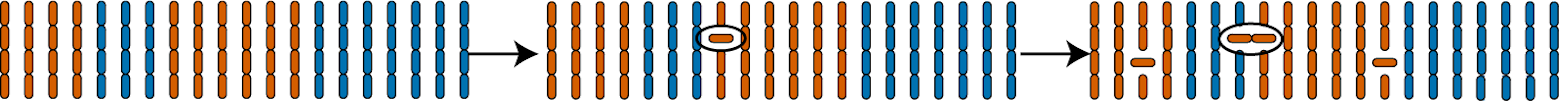

Supplement: S6 Fig — Fine-grained demonstration of how invasion occurs in the LM. (TIF) [file pcbi.1009381.s014.tif]

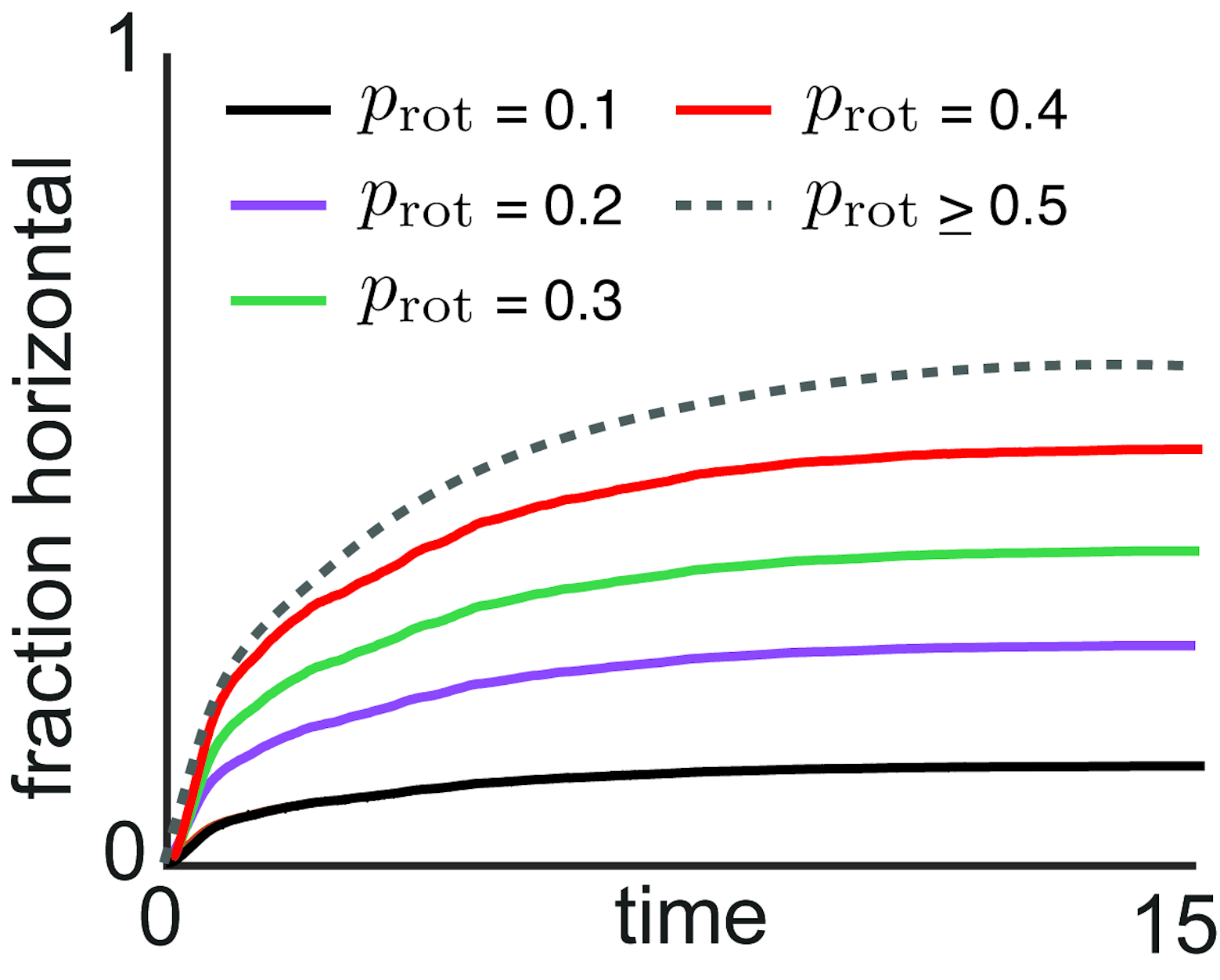

Supplement: S7 Fig — In the LM, a population of cells with a given prot value will reach an equilibrium value for the fraction of cells that are horizontal. For these simulations, we begin all cells vertically. We find that if prot is sufficiently large, then about half the population will be horizontal at any given time. (TIF) [file pcbi.1009381.s015.tif]

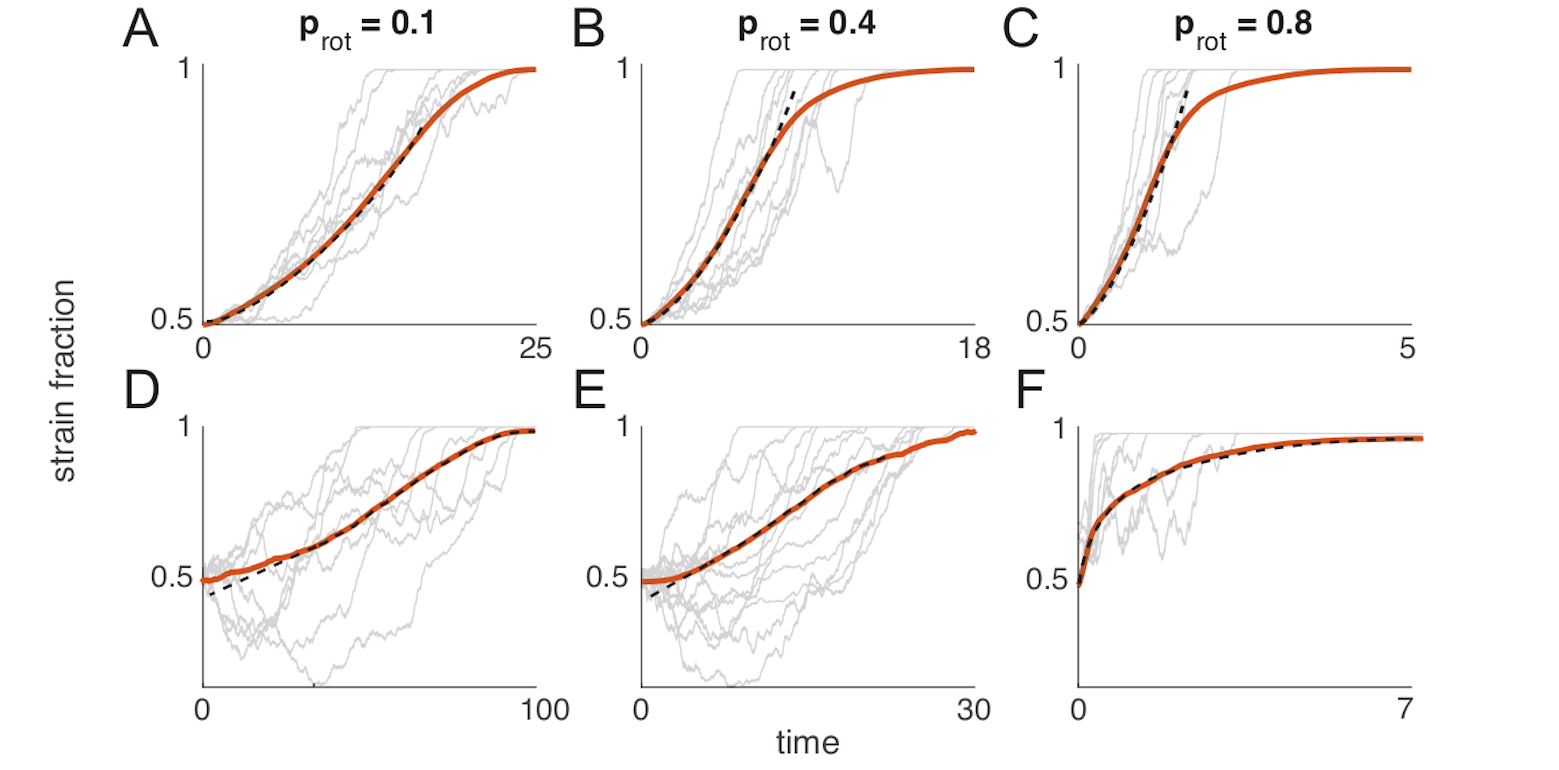

Supplement: S8 Fig — Further illustrations of invasion and bulk forcing in the LM. Top row: bulk forcing. Bottom row: invasion. For distinct prot values, we average several trajectories to obtain an average strain fraction temporal dynamics. We then fit exponential curves to the average time series to obtain the results seen in the insets of Fig 4 of the main text. For the top row, we fit the average dynamics to a function of the form 0.5eαt. For the bottom row, we fit the average dynamics to a function of the form 0.5(1 − e−αt). The resulting α versus 1 − prot relations can be seen in the insets of Fig 4. (TIF) [file pcbi.1009381.s016.tif]

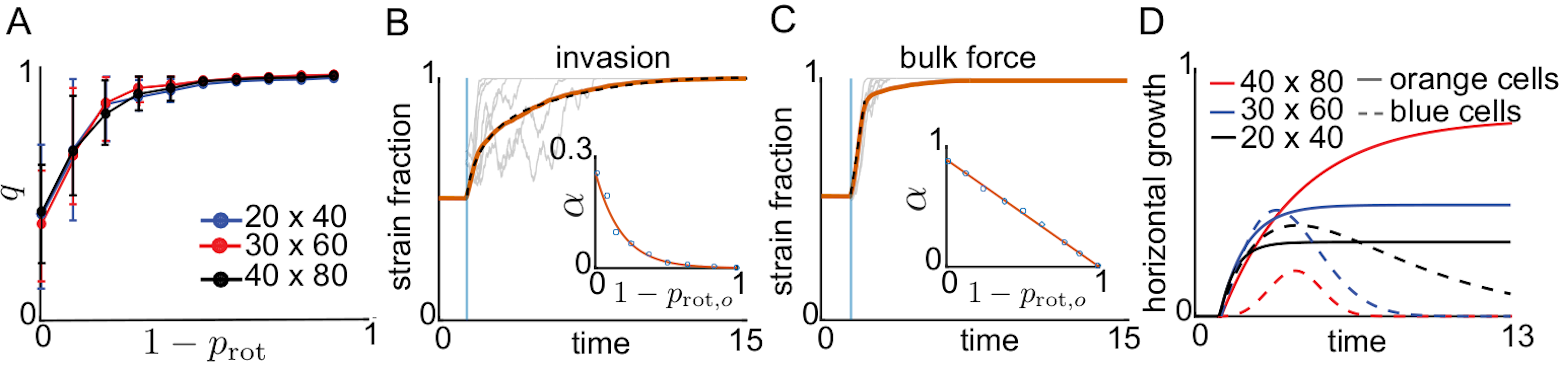

Supplement: S9 Fig — A replica of Fig 4 from the main text, but with q and α now plotted against 1 − prot. The trends here more directly mirror the trends shown in the ABM. (TIF) [file pcbi.1009381.s017.tif]
